# Integrative multi-omic analysis identifies genetically influenced DNA methylation biomarkers for breast and prostate cancers

**DOI:** 10.1038/s42003-022-03540-4

**Published:** 2022-06-16

**Authors:** Anita Sathyanarayanan, Hamzeh M. Tanha, Divya Mehta, Dale R. Nyholt

**Affiliations:** 1 Queensland University of Technology, Centre for Genomics and Personalised Health, Faculty of Health, Kelvin Grove, QLD Australia; 2 Queensland University of Technology, School of Biomedical Sciences, Faculty of Health, Kelvin Grove, QLD Australia

**Keywords:** Breast cancer, Cancer epigenetics, Computational biology and bioinformatics, Cancer genetics, Prostate cancer

## Abstract

Aberrant DNA methylation has emerged as a hallmark in several cancers and contributes to risk, oncogenesis, progression, and prognosis. In this study, we performed imputation-based and conventional methylome-wide association analyses for breast cancer (BrCa) and prostate cancer (PrCa). The imputation-based approach identified DNA methylation at cytosine-phosphate-guanine sites (CpGs) associated with BrCa and PrCa risk utilising genome-wide association summary statistics (*N*_BrCa_ = 228,951, *N*_PrCa_ = 140,254) and prebuilt methylation prediction models, while the conventional approach identified CpG associations utilising TCGA and GEO experimental methylation data (*N*_BrCa_ = 621, *N*_PrCa_ = 241). Enrichment analysis of the association results implicated 77 and 81 genetically influenced CpGs for BrCa and PrCa, respectively. Furthermore, analysis of differential gene expression around these CpGs suggests a genome-epigenome-transcriptome mechanistic relationship. Conditional analyses identified multiple independent secondary SNP associations (*P*_*cond*_ < 0.05) around 28 BrCa and 22 PrCa CpGs. Cross-cancer analysis identified eight common CpGs, including a strong therapeutic target in *SREBF1* (17p11.2)—a key player in lipid metabolism. These findings highlight the utility of integrative analysis of multi-omic cancer data to identify robust biomarkers and understand their regulatory effects on cancer risk.

## Abstract (web summary)

Methylome-wide association studies identify genetically-influenced CpGs associated with breast and prostate cancer risk and (epi)genome-transcriptome mechanistic relationships, with lipid metabolism genes implicated as potential therapeutic targets.

## Introduction

Breast cancer (BrCa) and prostate cancer (PrCa) are common cancers in women and men, respectively. Globally, BrCa accounted for 15% of cancer-related deaths in women while PrCa accounted for 6.7% of cancer-related deaths in men in 2018^1^. Despite occurring in different organs and sexes, they share roughly similar lifetime risks, hormonal involvement, and genetic factors in oncogenesis^2–4^. In addition, a family history of BrCa is associated with PrCa risk, and vice versa^5,6^. With the increasing incidence of these cancers, it is crucial to identify effective biomarkers and understand the underlying molecular similarities.

DNA methylation is an epigenetic mechanism that includes the addition of a methyl group to 5’ cytosine at cytosine-phosphate–guanine sites (CpGs). It regulates gene expression (e.g., DNA methylation in promoter regions correlates negatively with gene expression), chromatin structure formation, alternative splicing of mRNA precursors and normal mammalian development^7^. Aberrant DNA methylation has emerged as a hallmark in several cancers contributing to risk, oncogenesis, progression, and prognosis^8–10^. In BrCa, distinct DNA methylation patterns have been associated with molecular subtypes, oestrogen receptor status, germline *BRCA1* pathogenic variation, and prognosis^11,12^. Similarly, in PrCa, distinct DNA methylation signatures are observed among benign, primary, and metastatic prostate tissues, as well as subtypes of PrCa^13–15^.

DNA sequence variants, such as single-nucleotide polymorphisms (SNPs), have been shown to affect DNA methylation levels at CpGs. These variants are known as DNA methylation quantitative trait loci (meQTLs). MeQTL SNPs have been associated with the risk of numerous cancers, including breast and prostate cancers^16^. A recently developed approach—imputation-based methylome-wide association study (i-MeWAS)—integrates meQTL SNP information and results from genome-wide association studies (GWAS) to predict methylation of genetically influenced CpGs associated with the GWAS disease^17^. The approach imputes the methylation levels for a disease-associated dataset based on individual-level genotype data utilising meQTL SNP-based prediction models. The prediction models are generated using genotype and methylation measurements obtained from the same healthy individuals. Next, the associations of the imputed methylation levels with the disease are tested to find significant CpG associations. Methylation imputation and association testing can also be performed using GWAS summary statistics (GWAS-SS). Integrated analyses of meQTL SNPs and GWAS SNPs through approaches such as i-MeWAS aid the discovery of novel CpG biomarkers by leveraging the power of large GWAS and help ascertain the functional consequence of GWAS SNPs. Furthermore, by focusing on the genetically influenced CpG methylation associations, the approach limits biases due to confounding effects of the disease, medication, environmental effects and reverse causation on methylation levels, thereby providing robust biomarkers.

In this study, we propose a bioinformatics pipeline combining the i-MeWAS and conventional methylation association approach to identify an enriched set of genetically influenced CpGs associated with BrCa, PrCa and both cancers. For the genes associated with the identified CpGs, we perform differential gene expression analysis using TCGA datasets to explore the mechanistic link between the omic layers and gain further insights into the biological functions through pathway analysis. Lastly, through conditional analysis, we investigate the meQTL SNPs of the implicated CpGs for novel ‘secondary’ association signals.

## Results

### Genetically influenced and differentially methylated CpGs in individual cancers

**Fig. 1:**
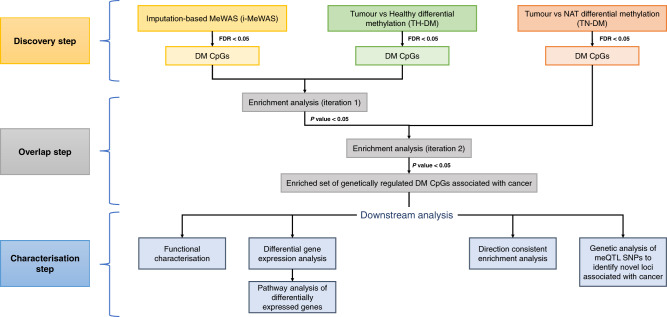
Bioinformatics pipeline to identify cancer-associated genetically influenced DNA methylation biomarkers (CpGs). The pipeline involves three steps including imputation-based and conventional methylome-wide association analyses using GWAS summary statistics and TCGA datasets, respectively, in the discovery step, enrichment testing and identification of an enriched set of cancer-associated CpGs in the overlap step, and lastly, functional characterisation of the CpGs, differential expression and pathway analysis of the genes associated to the CpGs, and genetic analyses in the characterisation step. CpGs cytosine-phosphate-guanine sites, DM differentially methylated, MeWAS methylome-wide association study, NAT histologically normal tissue adjacent to the tumour.

We developed a three-step bioinformatics pipeline based on blood and tumour tissues to detect genetically influenced differentially methylated CpGs associated with cancer (Fig. 1). The discovery step includes three differential methylation analyses using (i) cancer GWAS-SS and meQTL-based genetic prediction models (i-MeWAS), (ii) tumour and healthy methylation samples (TH-DM) and (iii) tumour and histologically normal adjacent to the tumour (NAT) methylation samples (TN-DM). While i-MeWAS identifies the differentially methylated (DM) CpGs in blood by imputation, the latter approaches identify the DM CpGs by comparing the observed measurements between tumour and control tissues using linear regression. The overlap step involves stepwise enrichment analysis to find a significant set of overlapping DM CpGs across the differential methylation analyses and are designated as cancer-associated CpGs. The characterisation step involves the characterisation of cancer-associated CpGs via differential gene expression, the directional effect of differential methylation, functional enrichment and conditional analyses.

**Fig. 2:**
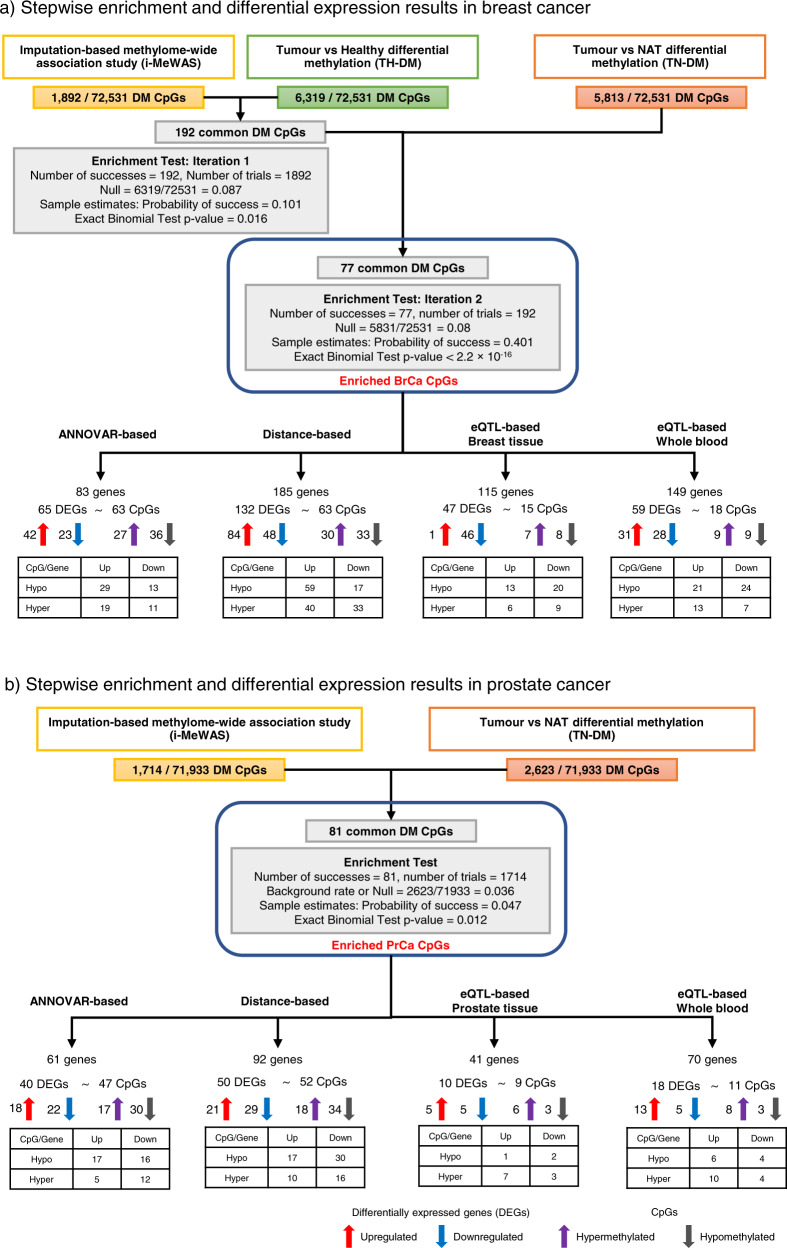
Identification of cancer-associated CpGs and differential expression analyses of the associated genes. Results from the overlap step of the pipeline and differential expression analyses of the genes associated with the enriched CpGs in (**a**) breast cancer (BrCa) and (**b**) prostate cancer (PrCa). Due to the unavailability of healthy prostate methylation data, the overlap step in PrCa did not include differential methylation between tumour and healthy tissues. For differential expression analysis, the CpGs were annotated to genes through ANNOVAR-, distance- and eQTL-based approaches. The differential expression was conducted in the TCGA gene expression dataset using DESeq2 for ANNOVAR- and distance-based genes and using the GWAS summary statistics and FUSION for eQTL-based genes. Up and Down for gene in the matrix refer to up- and downregulated gene expression while Hypo and Hyper for CpGs indicate hypo- and hyper-methylation, respectively. CpGs cytosine-phosphate–guanine sites, DEGs differentially expressed genes, DM differentially methylated, NAT histologically normal tissue adjacent to the tumour.

For BrCa analysis, we used the GWAS-SS from ref. ^18^. The DNA methylation data for 499 tumours and 91 NAT samples, and 81 healthy samples, collected by TCGA^19^, and GEO GSE101961^20^, respectively, were downloaded from EWAS Data Hub^21^. We tested differential methylation of 72,531 genetically influenced CpGs and detected 1892, 6319 and 5831 DM CpGs in the i-MeWAS, TH-DM and TN-DM analyses, respectively (FDR < 0.05). Stepwise enrichment identified 77 DM CpGs associated with BrCa (referred to as BrCa CpGs) (Fig. 2a and Supplementary Data 1). Of these, 22 and 10 CpGs were hypo- and hypermethylated, respectively, across all differential methylation analyses. Although not individually significant, when jointly examined we observed a significant enrichment of BrCa CpGs in the *5’-UTR*, *3’-UTR*, and *intronic* regions (*5’-UTR* + *intronic* + *3’-UTR*; binomial test: observed = 58.44%, expected = 44.48%, *P* = 0.016; Supplementary Data 2). We also observed a significant depletion of BrCa CpGs in the 1 kb region upstream of the gene transcription start site compared to the tested set of CpGs (binomial test: observed = 3.90%, expected = 15.81%, *P* = 0.002; Supplementary Data 2). Based on independent linkage disequilibrium (LD) blocks of the human genome defined in ref. ^22^, the 77 BrCa CpGs were distributed across 58 distinct LD blocks. Although most of the associated CpGs were located in distinct LD blocks, five LD blocks contained three CpGs, and nine LD blocks contained two CpGs. Functional characterisation performed using eForge v2^23^ identified that the BrCa CpGs were enriched with H3K4me1 histone marks in the majority of the tissue types, including blood (*P* = 1.64 × 10^−5^), breast (*P* = 1.13 × 10^−3^) and ovary (*P* = 7.19 × 10^−7^) (Supplementary Fig. 3).

For PrCa analysis, we used the GWAS-SS from ref. ^24^, and the DNA methylation data for 189 prostate tumour and 82 NAT samples collected by TCGA^13^ and GEO GSE76938^25^ were downloaded from EWAS Data Hub^21^. Due to the small number of healthy prostate samples with methylation data available, the discovery step included only i-MeWAS and TN-DM analyses. We tested 71,933 genetically influenced CpGs and identified 1714 and 2623 DM CpGs in the i-MeWAS and TN-DM analyses, respectively (FDR < 0.05). The overlap step identified 81 PrCa associated DM CpGs (referred to as PrCa CpGs) (Fig. 2b and Supplementary Data 3). There were 25 and 17 CpGs hypo- and hypermethylated, respectively, in both differential methylation analyses. There was a marginally significant increase in the proportion of PrCa CpGs in *upstream* (*P* = 0.091), *ncRNA intronic* (*P* = 0.078) and *5’-UTR* + *intronic* + *3’-UTR* (*P* = 0.074) regions and a near significant decrease in *intergenic* region (*P* = 0.051) compared to the set of all CpGs tested (Supplementary Data 4). With respect to LD blocks, the 81 PrCa CpGs were distributed across 46 distinct LD blocks. We observed one LD block containing eight CpGs, one LD block containing six CpGs, two LD blocks containing four CpGs, three LD blocks containing three CpGs, 11 LD blocks containing two CpGs, and the remaining 28 CpGs in distinct LD blocks. Functional characterisation showed that the PrCa CpGs were significantly enriched with H3K4me1 histone marks in most of the tissue types including blood (*P* = 8.79 × 10^−7^) and breast (*P* = 3.34 × 10^−4^) (Supplementary Fig. 4).

### Differential methylation influencing gene expression

To determine if the DM CpGs associated with cancer were involved in transcription regulation, we tested for differential expression of the CpG-associated genes using TCGA tumour and NAT gene expression data. We used three approaches to define the genes associated with the CpGs: (i) ANNOVAR-based, (ii) distance-based and (iii) eQTL-based. For the ANNOVAR- and distance-based genes, differential expression was analysed using DESeq2^26^ and mediation analysis, while for eQTL-based genes, differential expression was analysed using FUSION software^27^. Lastly, we explored the biological functions of the differentially expressed genes (DEGs) via pathway analysis using the EnrichR^28^ webtool.

#### ANNOVAR-based

To identify the CpG-associated DEGs, we annotated the CpGs to genes using ANNOVAR followed by differential gene expression analysis. In BrCa, the 77 BrCa CpGs were annotated to 83 genes by ANNOVAR^29^ which had valid measurements in the TCGA BrCa gene expression dataset. We identified 65 DEGs (FDR < 0.05) (Fig. 2a and Supplementary Data 5), of which 42 were upregulated (mean fold change =  1.76) and 23 were downregulated (mean fold change = 0.45). We investigated the relationship between methylation and gene expression by comparing the direction of differential expression of the DEGs with that of the differential methylation of the associated BrCa CpGs in the TN-DM analysis. The 65 DEGs were associated with 63 BrCa CpGs, forming 72 unique CpG-gene pairs. A negative correlation was observed in 40 CpG-gene pairs. For example, the CpGs in *CDC7*, *MICAL2* and *MUC1* were hypomethylated, while the genes were upregulated. In contrast, a positive correlation was observed in 32 CpG-gene pairs where both the methylation and the gene expression either increased or decreased in tumour samples compared to NAT samples (Fig. 2a and Supplementary Data 5). Following this, we conducted a conservative mediation analysis to examine the conditional differential expression of the genes given the methylation status of the associated CpG. We identified 14 genes to be differentially expressed after conditioning for CpG methylation (conditional association FDR < 0.05; Supplementary Data 6). The 14 DEGs were associated with 13 BrCa CpGs and formed 14 CpG-gene pairs.

The PrCa CpGs were annotated to 61 genes by ANNOVAR and differential expression analysis in TCGA PrCa gene expression dataset detected 40 DEGs (FDR < 0.05). Of these, 18 were upregulated (mean fold change = 1.70) and 22 were downregulated (mean fold change = 0.53) (Fig. 2b and Supplementary Data 7). The 40 DEGs were associated with 47 CpGs and formed 50 unique CpG-gene pairs, of which 29 CpG-gene pairs exhibited a negative correlation between methylation and gene expression and 21 CpG-gene pairs exhibited a positive correlation. Next, we conducted the meditation analysis which identified four genes with significant evidence for differential expression after conditioning for CpG methylation (conditional association FDR < 0.05; Supplementary Data 8). The four DEGs were associated with four CpGs and formed six CpG-gene pairs.

#### Distance-based

To identify the CpG-associated DEGs, we first estimated the proportion of DEGs in the flanking regions of varying lengths from 1 kb to 10 Mb around a CpG. This was followed by the selection of the flanking region that showed the maximum decrease in the proportion of DEGs when increasing the region length. The DEGs of the selected region were assigned as the CpG-associated DEGs.

In BrCa, we selected the 132 DEGs in the ±25 kb flanking window as the likely implicated DEG set (~6% decrease was observed when increasing the window size from 25 to 50 kb, Supplementary Data 9 and 10). Mediation analysis of the genes in the ±25 kb flanking window of the BrCa CpGs found 26 DEGs after conditioning for CpG methylation (conditional association FDR < 0.05; Supplementary Data 11). In PrCa, the maximum decrease in the proportion of DEGs (~11%) was seen when increasing the flanking region from 10 kb (54.35%) to 25 kb (43.75%) and the 50 DEGs in the ±10 kb flanking window were chosen as the implicated DEG set (Supplementary Data 12 and 13). Mediation analysis of the genes ±10 kb flanking window provided evidence for one gene to be differentially expressed given the CpG methylation (conditional association FDR < 0.05; Supplementary Data 13).

#### eQTL-based

The CpG-associated DEGs were selected as those genes that were differentially expressed as well as genetically influenced by the same SNPs that influence the enriched CpGs. To identify the genes genetically influenced by the same SNPs as the enriched CpGs, gene expression prediction models developed based on expression quantitative trait loci (eQTL) SNPs for the genes were downloaded from FUSION^27^. Next, those genes with significant enrichment of the meQTL SNPs (based on binomial tests) in their expression prediction models were selected. To identify the CpG-associated DEGs, we performed an imputation-based differential expression analysis for the selected genes using FUSION^27^. As SNP-based expression prediction models are available for individual tissues, we examined the genetically influenced genes in whole blood and breast tissue for BrCa, and whole blood and prostate tissue for PrCa.

In BrCa, we identified 115 and 149 genes in breast tissue and whole blood, respectively, with significant enrichment of meQTL SNPs in their prediction models (FDR < 0.05). Of these, 47 and 59 genes in breast tissue and whole blood, respectively, were differentially expressed (FDR < 0.05; Supplementary Data 14). A total of 46 of the 47 DEGs in breast tissue were downregulated, while 31 of the 59 DEGs were upregulated in whole blood. In PrCa, 41 and 52 genes in prostate tissue and whole blood, respectively, were significantly enriched with meQTL SNPs in their prediction models (FDR < 0.05). Of these, 10 and 18 were differentially expressed in prostate tissue and whole blood, respectively (Supplementary Data 15). In prostate tissue five of the 10 DEGs were upregulated, while in whole blood 13 of the 18 DEGs were upregulated.

#### Pathway analysis

We identified the pathways associated with the DEGs using EnrichR^28^. In BrCa, the pathways associated with ANNOVAR-based DEGs were broadly related to cancer, cell differentiation and cellular processes such as cell communication, junction and adhesion, as well as hormonal regulation (Table 1). The distance-based DEGs were associated with pathways related to cancer, DNA repair and cell death and the eQTL-based genes were associated with an endocrine system and lipid metabolism pathway. In PrCa, the pathways associated with ANNOVAR-based and distance-based DEGS displayed substantial overlap (Table 2). These pathways were related to lipid metabolism, laminopathy and apoptosis. Pathway analysis of eQTL-based genes did not identify any significant pathways.

**Table 1:** Pathways of ANNOVAR-, distance- and eQTL-based differentially expressed genes associated with breast cancer CpGs.

Gene association approach	Pathway database	Pathway	FDR
ANNOVAR-based	BioPlanet	Cell–cell communication	2.44 × 10^−3^
		Cell junction organisation	6.13 × 10^−3^
		Cell adhesion molecules (CAMs)	3.42 × 10^−2^
		Circadian rhythm	3.89 × 10^−2^
	Elsevier	Oestrogen deficiency in female obesity	6.64 × 10^−3^
		Circadian clock in sleep regulation	2.92 × 10^−2^
		Telogen maintenance in androgenic alopecia	4.03 × 10^−2^
		Local oestrogen production in endometriosis	4.46 × 10^−2^
	KEGG	Cell adhesion molecules	2.20 × 10^−2^
		Tight junction	3.35 × 10^−2^
	MSigDB	UV response Dn	1.12 × 10^−2^
		Mitotic spindle	3.21 × 10^−2^
		Oestrogen response early	3.21 × 10^−2^
		Apical junction	3.21 × 10^−2^
	Reactome	Cell–cell communication	6.87 × 10^−3^
		Cell junction organisation	1.78 × 10^−2^
Distance-based	BioCarta	Role of *BRCA1*, *BRCA2* and *ATR* in cancer susceptibility	1.83 × 10^−2^
		Caspase cascade in apoptosis	1.83 × 10^−2^
	Elsevier	Oestrogen deficiency in female obesity	9.40 × 10^−4^
eQTL-based	WikiPathway	Glycerolipids and glycerophospholipids	2.04 × 10^−2^
		Fatty acid beta-oxidation	3.83 × 10^−2^

**Table 2:** Pathways of ANNOVAR- and distance-based differentially expressed genes associated with prostate cancer CpGs.

Gene association approach	Pathway database	Pathway	FDR
ANNOVAR	BioCarta	Caspase cascade in apoptosis	9.61 × 10^−4^
	Elsevier	Lipodystrophy, familial partial	2.03 × 10^−3^
		Familial partial lipodystrophy type 2 progression (hypothesis)	2.82 × 10^−3^
		Hutchinson-Gilford Progeria syndrome	3.12 × 10^−3^
		Nuclear lamina cleavage	3.62 × 10^−3^
		Nuclear envelope in cell division	3.62 × 10^−3^
		mTOR signalling	3.43 × 10^−2^
	KEGG	Glutathione metabolism	2.90 × 10^−2^
	Reactome	Detoxification of reactive oxygen species	2.97 × 10^−2^
	WikiPathway	The influence of laminopathies on Wnt signalling	1.34 × 10^−2^
		The overlap between signal transduction pathways that contribute to a range of LMNA laminopathies	2.71 × 10^−2^
Distance	BioCarta	Caspase cascade in apoptosis	1.50 × 10^−3^
	Elsevier	Lipodystrophy, familial partial	3.18 × 10^−3^
		Familial partial lipodystrophy type 2 progression (hypothesis)	4.41 × 10^−3^
		Hutchinson-Gilford Progeria syndrome	4.88 × 10^−3^
		Nuclear lamina cleavage	5.65 × 10^−3^
		Nuclear envelope in cell division	5.65 × 10^−3^
	WikiPathway	The influence of laminopathies on Wnt signalling	2.07 × 10^−2^
		The Overlap between signal transduction pathways that contribute to a range of LMNA laminopathies	4.18 × 10^−2^

Note: In PrCa, pathway analysis of the eQTL-based DEGs did not identify any significant pathways.

### Directional effect of differential methylation in individual cancers

Given global hypomethylation and site-specific hypermethylation have been reported in breast and prostate cancers^30–32^, we examined the association of hypo- and hypermethylated CpGs with each cancer. For this, we selected the nominally significant hypo- or hypermethylated CpGs (uncorrected *P* < 0.05) in the discovery step and conducted the overlap step in the pipeline.

In both cancers, we did not find evidence for significant genome-wide hypermethylation (Supplementary Data 16). However, the site-specific analysis identified hypermethylated CpGs enriched in *5’-UTR*, *exonic*, *3’-UTR*, *downstream*, *ncRNA exonic*, and *ncRNA intronic* regions in BrCa, and *3’-UTR* region in PrCa (Supplementary Data 17).

Concerning hypomethylation, significant genome-wide hypomethylation was observed in BrCa (*P* < 1 × 10^−16^) and an enriched set of 298 hypomethylated CpGs associated with BrCa were identified (Supplementary Data 16). To explore the potential biological implication of genome-wide hypomethylation, we identified the associated genes using ANNOVAR annotation followed by differential expression and pathway analyses. There were 218 DEGs (FDR < 0.05), including 128 upregulated genes (mean fold change = 2.36) and 90 downregulated genes (mean fold change = 0.56). The pathway analysis of 218 DEGs using EnrichR^28,33^ webtool identified 141 pathways that were related to the endocrine system, lipid metabolism, signal transduction and cancer (Supplementary Data 18). In PrCa, there were 400 overlapping hypomethylated CpGs between i-MeWAS and TN-DM showing marginally significant enrichment (*P* = 0.068, Supplementary Data 16). It is possible that the unavailability of a sufficient number of tumour methylation samples, as well as healthy methylation samples, could have decreased the statistical power to find an enriched set of hypomethylated CpGs in PrCa. However, the current results from directional analysis support previous findings^30–32^ that there is genome-wide hypomethylation in PrCa. There were 184 significant DEGs (FDR < 0.05) associated with these 400 hypomethylated CpGs, comprising 93 upregulated genes (mean fold change = 1.76) and 91 downregulated genes (mean fold change = 0.56). The DEGs were significantly involved in seven pathways including transcription regulation and oestrogen response (FDR < 0.05, Supplementary Data 18).

### Genetic analysis of the meQTLs associated with BrCa and PrCa CpGs

Having characterised the enriched CpGs and the associated genes, we next investigated the associated meQTL SNPs. The 77 BrCa CpGs were influenced by 3040 unique meQTL SNPs and the 81 PrCa CpGs were influenced by 3049 unique meQTL SNPs. We estimated the contribution of these meQTL SNPs to familial relative risk (FRR) of BrCa and PrCa using the method provided in ref. ^18^, and the BrCa GWAS-SS and PrCa GWAS-SS, respectively. We estimated that the 3040 meQTL SNPs associated with the BrCa CpGs explain 36.25% of the assumed overall BrCa FRR of 2^18^, while the 3049 meQTL SNPs associated with the PrCa CpGs explain 86.92% of the assumed PrCa FRR of 2.5^34^.

**Fig. 3:**
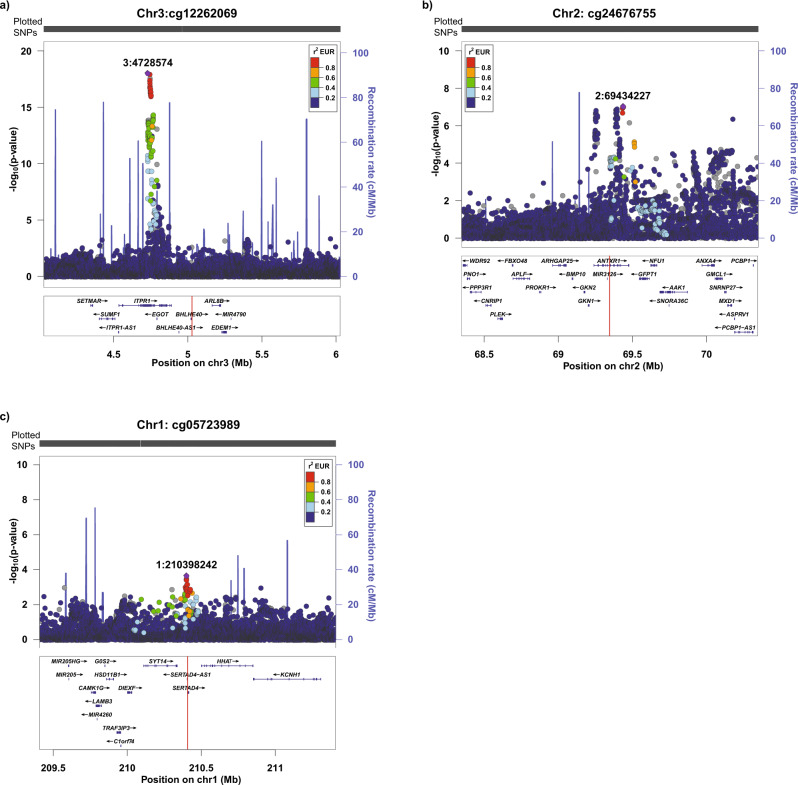
LocusZoom plots of the ±1 Mb flanking regions for selected breast cancer (BrCa) CpGs. The red line in the gene panel indicates the genomic location of the CpG site. Panel **a** is an example of a BrCa CpG with genome-wide significant SNPs in the flanking region, panel **b** is an example of a BrCa CpG with suggestive SNPs in the flanking region, and panel **c** is an example of a BrCa CpG identified in a novel region (i.e., no genome-wide significant or suggestive SNPs present in the flanking region).

As the i-MeWAS approach predicts the effects of CpG methylation on a disease by considering the effects of SNPs on CpG methylation and disease, we examined the GWAS association of SNPs in ±1 Mb flanking genomic regions around the associated cancer CpGs. Among the 77 BrCa CpGs, 37 had at least one genome-wide significant SNP (*P*_*GWAS*_ ≤ 5 × 10^−8^) in the flanking regions, 25 had at least one suggestive SNP (5 × 10^−8^ < *P*_*GWAS*_ ≤ 1 × 10^−5^), and 15 were present in novel genomic regions—i.e., the flanking regions had no genome-wide significant or suggestive SNPs (Fig. 3 and Supplementary Data 19). Among the 81 PrCa CpGs, we identified 48, 28 and 5 PrCa CpGs in genome-wide significant, suggestive and novel genomic regions, respectively (Supplementary Data 20).

We hypothesised that the CpGs in the novel and suggestive genomic regions were significant in our enrichment pipeline because, among the predictor meQTL SNPs for such CpGs, there may be multiple independent signals that jointly contribute to cancer via methylation regulation. To test this, we performed an iterative conditional analysis of the meQTL SNPs using the conditional and joint (COJO) analysis function from the genome-wide complex trait analysis (GCTA) software (version 1.93.2)^35^. We start the iteration by conditioning on the meQTL SNP with the most significant cancer GWAS association (*P*_*GWAS*_). Next, we select the meQTL SNP with the smallest conditional *P* (*P*_*cond*_) value less than the cut-off *P* = 0.05. We then perform conditional analysis incorporating the newly selected meQTL SNP. We repeat the steps until no new meQTL SNP satisfies the selection criteria (*P*_*cond*_ < 0.05). The selected meQTL SNPs are interpreted as being associated with cancer via their effect on DNA methylation at CpGs.

In BrCa, stepwise conditional analysis of the meQTL SNPs of CpGs in suggestive and novel regions found evidence for multiple independent meQTL SNPs for 28 CpGs at *P*_*cond*_ < 0.05 associated with BrCa (Supplementary Data 21). Of note are the independent meQTL SNPs of cg14494596 (3q21.31), cg03958883 (11q13.4) and cg16555866 (17q25.3) as they showed increased evidence for association after stepwise conditional analysis (Table 3). For 11 CpGs, the conditional analysis found no secondary meQTL SNPs (*P*_*cond*_ < 0.05) after the first iteration indicating that a single meQTL SNP was driving the association of the CpG with BrCa risk. Of these 11 single meQTL SNPs, seven were suggestive or very close to suggestive significance (5 × 10^−8^ < *P*_*GWAS*_ < 8 × 10^−5^) while the remaining meQTL SNPs were nominally significant (8 × 10^−5^ < *P*_*GWAS*_ < 2 × 10^−3^). In PrCa, the stepwise conditional analysis identified multiple independent meQTL SNPs for 22 CpGs associated with PrCa (Supplementary Data 22). For eight CpGs on 17p11.2 that were analysed together, we identified seven significant meQTL SNPs after conditional analysis. All the meQTL SNPs showed an increase in association with PrCa risk after conditional analysis (Table 4). For CpGs where conditional analysis found no secondary meQTL SNPs, the single meQTL SNP was either suggestive or close to suggestive (*P*_*GWAS*_ < 5 × 10^−4^). Taken together, the significant evidence of residual association and low linkage disequilibrium (LD) among these meQTL SNPs support these to be true independent secondary associations jointly implicated in cancer via their effect on DNA methylation at CpGs.

**Table 3:** Significant secondary associations identified around selected breast cancer CpGs through conditional analysis of predictor meQTL SNPs.

CpG	Chr	Location (bp)	Cytoband	meQTL SNP	Location (bp)	Effect allele	Breast cancer GWAS results			Conditional analysis, LD from 1000 G phase 3 (EUR)
							Frequency	β	*P*_*GWAS*_	*P*_*cond*_^a^
cg14494596	3	48542040	3q21.31	rs34071734	49425004	A	0.061	−0.042	1.56 × 10^−3^	–
				rs767512	48023924	C	0.290	−0.018	9.24 × 10^−3^	4.95 × 10^−3^
				rs13095891	48381826	T	0.053	−0.036	1.15 × 10^−2^	5.93 × 10^−3^
				rs112445131	47786930	T	0.175	−0.012	1.51 × 10^−1^	4.59 × 10^−3^
				rs3895736	48658467	A	0.172	−0.013	1.20 × 10^−1^	1.70 × 10^−3^
				rs73078357	48695834	C	0.126	−0.014	1.45 × 10^−1^	1.36 × 10^−2^
cg03958883	11	73020729	11q13.4	rs11235743	73124826	G	0.216	0.023	2.59 × 10^−3^	–
				rs11602616	72364077	C	0.166	0.025	4.34 × 10^−3^	7.24 × 10^−3^
				rs7116276	72276606	T	0.315	0.013	5.76 × 10^−2^	1.68 × 10^−2^
				rs77383504	72265531	G	0.055	0.024	1.13 × 10^−1^	1.95 × 10^−2^
				rs11235541	72285444	A	0.046	0.017	3.12 × 10^−1^	4.50 × 10^−2^
				rs61893925	72274449	T	0.111	0.019	5.88 × 10^−2^	3.59 × 10^−2^
cg16555866	17	80830922	17p11.2	rs78483419	80870884	C	0.180	−0.032	7.78 × 10^−5^	–
				rs8076573	80496771	G	0.712	0.025	1.62 × 10^−3^	4.98 × 10^−4^
				rs78165269	80601498	C	0.304	−0.024	8.72 × 10^−4^	2.79 × 10^−3^
				rs4789841	80739952	T	0.073	−0.024	6.73 × 10^−2^	1.07 × 10^−2^

*Chr* chromosome.

^a^At each CpG site, the *P*_*cond*_ for a meQTL SNP is the association significance after conditioning on all the meQTL SNPs present in the rows above it. For example, the *P*_*cond*_ of rs7116276 is calculated after conditioning on rs11235743 and rs11602616.

**Table 4:** Significant secondary associations identified around selected prostate cancer CpGs through conditional analysis of predictor meQTL SNPs.

CpG	Chr	Location (bp)	Cytoband	meQTL SNP	Location (bp)	Effect allele	Prostate cancer GWAS results			Conditional analysis, LD from 1000 G phase 3 (EUR)
							Frequency	β	*P*_*GWAS*_	*P*_cond*_
cg14752965, cg14720773, cg27208169, cg06775073, cg17844831, cg19447962, cg10870160, cg08129017	17	17579346, 17603531, 17603584, 17603749, 17604146, 17628656, 17695459, 17728660		rs9899634	17727943	A	0.637	−0.040	9.47 × 10^−7^	–
				rs55888125	18505676	T	0.519	−0.024	7.41 × 10^−3^	6.79 × 10^−3^
				rs7225876	17178553	T	0.658	−0.019	2.97 × 10^−2^	3.96 × 10^−3^
			17p11.2	rs75534953	17300299	A	0.053	−0.046	3.60 × 10^−2^	7.91 × 10^−3^
				rs4641803	16936194	T	0.124	−0.025	5.83 × 10^−2^	3.20 × 10^−3^
				rs77287723	17508656	C	0.081	0.018	2.26 × 10^−1^	2.05 × 10^−2^
				rs8079130	16884960	T	0.142	0.029	1.13 × 10^−2^	3.99 × 10^−2^

*Chr* chromosome.

*At each CpG site, the *P*_*cond*_ for a meQTL SNP is the association significance after conditioning on all the meQTL SNPs present in the rows above. For example, the *P*_*cond*_ of rs4641803 is calculated after conditioning on rs9899634, rs55888125, rs7225876 and rs75534953.

For the CpGs in genome-wide significant regions (*N*_*BrCa*_ = 37, *N*_*PrCa*_ = 48), we hypothesised that the genome-wide significant SNPs in the region are mediating the cancer risk effects via the meQTL SNPs and their effect on DNA methylation at CpGs. Hence, we estimated the LD between the meQTL SNPs and the genome-wide significant SNPs using the 1000 Genome Phase 3 for the European population as a reference panel and LDlinkR R package^36^. We found 28 and 19 CpGs in the genome-wide significant regions in BrCa and PrCa, respectively—having at least one meQTL SNP in moderate to strong LD (*r*^*2*^ > 0.4) with at least one genome-wide significant SNP (Supplementary Data 23 and 24). These results indicate that (i) the association of these CpGs to BrCa or PrCa is likely due to their meQTL SNPs being in LD with BrCa or PrCa genome-wide significant SNPs, and (ii) the remaining CpGs with no strong LD with an individual genome-wide significant BrCa or PrCa GWAS SNP could suggest that the predictor SNPs are in LD with multiple secondary association signals that require more powerful GWAS to identify.

#### Pleiotropic genetically influenced and differentially methylated CpGs in breast and prostate cancers

Given the strong evidence for shared genetic risk factors between BrCa and PrCa, we investigated the presence of pleiotropic genetically influenced DM CpGs. To this end, we examined the implicated BrCa and PrCa CpGs that were annotated to the same genes. There were six common genes—*BRI3*, *LRATD2*, *PCAT1*, *LOC102724265*, *LINC01488* and *SREBF1*—that were associated with four BrCa and four PrCa CpGs, including one common CpG (cg08129017) in *SREBF1* (Table 5). To confirm if the remaining three BrCa and PrCa CpG pairs of each gene are pleiotropic, we investigated the LD between the meQTL SNPs associated with the BrCa CpG and that of the PrCa CpG. The presence of at least one pair of meQTL SNPs with at least moderate LD (*r*^*2*^ > 0.4) suggests that the CpGs are likely pleiotropic. We found that all three BrCa and PrCa CpG pairs were linked via moderate to strong LD between their predictor meQTL SNPs; thus indicating that these CpGs are likely pleiotropic genetically influenced and differentially methylated CpGs in breast and prostate cancers.

**Table 5:** Breast cancer (BrCa) and prostate cancer (PrCa) differentially methylated CpGs annotated to the same genes.

Gene	BrCa CpG	PrCa CpG	Common meQTL SNPs	LD of *r*^*2*^ > 0.4 between meQTL SNPs of BrCa and PrCa CpGs
*BRI3*	cg01877450	cg23245481	0	7 BrCa meQTL SNPs with 2 PrCa meQTL SNPs
*LRATD2*, *PCAT1*	cg16015285	cg03374695	2	4 BrCa meQTL SNPs with 17 PrCa meQTL SNPs
*LOC102724265, LINC01488*	cg08885142	cg18498241	2	16 BrCa meQTL SNPs with 16 PrCa meQTL SNPs
*SREBF1*	cg08129017	cg08129017	NA	NA

**Fig. 4:**
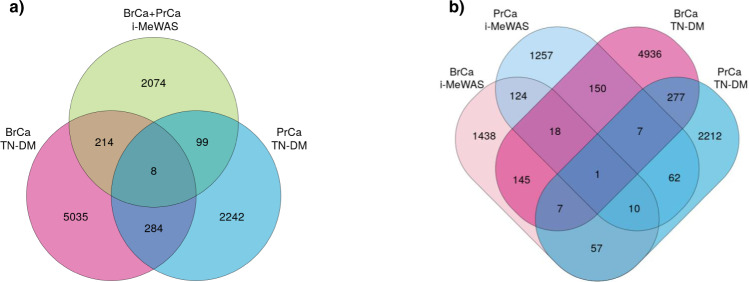
Cross cancer analysis to identify the pleiotropic DM CpGs between breast cancer (BrCa) and prostate cancer (PrCa). **a** Venn overlap of i-MeWAS results using BrCa and PrCa meta-analysed GWAS-SS (BrCa+PrCa i-MeWAS), differential methylation results of tumour vs NAT in BrCa (BrCa TN-DM), and differential methylation results of tumour vs NAT in PrCa (PrCa TN-DM). **b** Venn overlap of independent i-MeWAS results using BrCa GWAS-SS (BrCa i-MeWAS) and PrCa GWAS-SS (PrCa i-MeWAS), and independent differential methylation results of tumour vs NAT in BrCa (BrCa TN-DM) and PrCa (PrCa TN-DM). i-MeWAS, imputation-based methylome-wide association study.

To identify further pleiotropic DM CpGs, we conducted two similar but distinct overlap analyses: (i) overlap of i-MeWAS results based on meta-analysed BrCa and PrCa GWAS-SS with BrCa TN-DM and PrCa TN-DM results; and (ii) overlap of i-MeWAS results based on individual cancer GWAS-SS with BrCa TN-DM and PrCa TN-DM results. Both overlap analyses were based on 68,613 CpGs for which methylation measurements were available in all datasets. For the overlap analysis using the meta-analysed GWAS-SS, BrCa and PrCa GWAS-SS were combined using an inverse-variance weighted fixed-effect meta-analysis with the GWAMA software^37^. Meta-analysis of the GWAS-SS can increase the statistical power to identify pleiotropic genetic loci and consequently provide more information to predict the common genetically influenced DM CpGs in both cancers. i-MeWAS of the meta-analysed GWAS-SS (BrCa + PrCa i-MeWAS) identified 2395 DM CpGs, while the BrCa TN-DM and the PrCa TN-DM identified 5541 and 2633 DM CpGs, respectively (FDR < 0.05). Pairwise overlap showed significant enrichment between all pairs except BrCa+PrCa i-MeWAS and PrCa TN-DM (one-side binomial test *P* = 0.06; Supplementary Data 25). Overall, eight overlapping DM CpGs between the three DM analyses were identified (Fig. 4a and Table 6) with six CpGs exhibiting consistent differential methylation direction in all three analyses. We further examined the association significance of the SNPs present in the ±1 Mb flanking regions of the eight common CpGs in the meta-analysed BrCa+PrCa GWAS and found that two CpGs, cg07421287 on 1p13.3 near *KCNA3* and cg09205595 on 7q36.1 near *AGAP3*, were present in novel genomic locations—i.e., there were no genome-wide significant SNPs (*P*_*GWAS*_ < 5 × 10^−8^) in the flanking genomic regions (Supplementary Fig. 5). We also examined the eight regions in the individual BrCa and PrCa GWAS-SS and found three CpGs, cg07421287 (1p13.3), cg09205595 (7q36.1) and cg08129017 (17p11.2) were present in genomic regions with no genome-wide significant SNPs in both GWASs (Supplementary Data 26).

**Table 6:** Common differentially methylated CpGs in breast and prostate cancers identified using overlap analyses.

CpG	CHR	Location (bp)	Cytoband	Gene	BrCa+PrCa i-MeWAS Z score	BrCa i-MeWAS *Z* score	PrCa i-MeWAS *Z* score	BrCa TN-DM *Z* score	PrCa TN-DM *Z* score
cg07421287*	1	111218287	1p13.3	*KCNA3*	3.14	0.71	4.17	−4.97	−5.83
cg24789467*^#^	5	132159003	5q31.1	*SHROOM1*	−4.29	−2.99	−3.15	−4.13	−3.73
cg09205595*^#^	7	150782539	7q36.1	*AGAP3*	3.51	2.96	1.91	3.75	4.12
cg26405020^#^	15	91427363	15q26.1	*FES, FURIN*	−5.10	−4.03	−3.14	3.23	3.71
cg14918082*	17	7833237	17p13.1	*KCNAB3, TRAPPC1*	−3.73	−2.14	−3.30	−5.02	−4.78
**cg08129017**	**17**	**17728660**	**17p11.2**	***SREBF1***	**5.64**	**3.58**	**4.58**	**5.20**	**3.29**
cg20513976*	20	62367893	20q13.33	*LIME1*	−3.97	0.84	−7.71	−2.93	−5.21
cg12413156*	20	62368256	20q13.33	*LIME1*	−3.82	1.40	−8.20	−3.30	−3.74

*BrCa* breast cancer, *PrCa* prostate cancer, *i-MeWAS* imputation-based methylome-wide association study, *TN-DM* tumour vs normal tissue adjacent to tumour differential methylation.

*and ^#^indicate that the CpGs that were not significantly differentially methylated in BrCa i-MeWAS analysis (FDR > 0.05) PrCa i-MeWAS analysis (FDR > 0.05), respectively. The row in bold font indicates the common DM CpG between the eight DM CpGs identified through the overlap analysis using BrCa+PrCa meta-analysed GWAS-SS (Fig. 4a) and the one DM CpG identified through the overlap analysis of individual GWAS-SS (Fig. 4b).

While conducting a meta-analysis of the two cancers can increase the statistical strength of the SNPs, it assumes that a genetic variant (allele) has a homogeneous effect on the risk of both cancers. In the extreme example of a genetic variant having opposite effects on BrCa and PrCa risks, the effects would cancel each other out and produce no evidence for association in a meta-analysis. To overcome this potential issue, we performed the next overlap analysis using the DM CpGs from independent i-MeWAS analysis of BrCa and PrCa GWAS-SS. After correction for 68,613 tests, 1800 and 1629 were predicted as differentially methylated in the BrCa i-MeWAS and PrCa i-MeWAS, respectively (FDR < 0.05). The overlap of these results along with the BrCa and PrCa TN-DM results identified one common CpG (cg08129017) (Fig. 4b), which showed similar hypermethylation across all DM analyses (Table 6). It should be noted that the pairwise overlap was significant for all pairs except BrCa i-MeWAS and PrCa TN-DM (one-sided binomial test *P* = 0.25, Supplementary Data 25).

As the cg08129017 CpG was consistently identified across different analyses, we examined the associated meQTL SNPs and genes. We found that the meQTL SNPs showing similar nominal associations (1 × 10^−5^ < *P*_*GWAS*_ < 0.05) in the individual BrCa and PrCa GWAS-SS reached genome-wide significance (*P*_*metaGWAS*_ < 5 × 10^−8^) in the meta-analysed BrCa+PrCa GWAS-SS (Supplementary Data 27). The heterogeneity of SNP effects (Cochran’s Q) for each SNP was estimated using GWAMA software^37^ and displayed no significant heterogeneity at threshold *P*_*het*_ < 0.05 for nominal evidence. Stepwise conditional analysis of the meQTL SNPs in the meta-analysed BrCa+PrCa GWAS-SS provided evidence for two independent associations (rs2236513, *P*_*cond*_ = 6.41 × 10^−9^ and rs12943647, *P*_*cond*_ = 9.55 × 10^−3^; LD *r*^2^ = 0.0004) with both BrCa and PrCa. While cg08129017 was consistently hypermethylated across all analyses, differential expression analysis of the *SREBF1* gene annotated to this CpG by ANNOVAR showed a small but significant upregulation in both cancers. We observed a differential expression fold change of 1.26 (*P*_*DeSEQ2*_ = 4.12 × 10^−23^) and 1.41 (*P*_*DeSEQ2*_ = 1.46 × 10^−3^) for BrCa and PrCa, respectively (Supplementary Data 5 and 7). In addition, we sought to test the differential expression of *RAI1* and *TOM1L2* genes that were also located in the ±25 kb flanking region of this CpG and had valid gene expression information in both cancer datasets. However, these genes were differentially expressed only in BrCa (*RAI1* gene: fold change =  1.52, *P*_*DeSEQ2*_ = 3.48 × 10^−14^ and *TOM1L2* gene: fold change = 0.83, *P*_*DeSEQ2*_ = 2.98 × 10^−3^).

## Discussion

This study systematically tested the associations of genetically influenced DNA methylation at CpGs with BrCa and PrCa using GWAS-SS and experimental methylation datasets. We discovered 77 and 81 DM CpGs associated with BrCa and PrCa, respectively. Combined with differential gene expression and genetic analyses, we provide insight into the probable relationships between the different omics.

Our multi-omics study overcomes limitations faced by conventional GWAS analyses. First, detecting novel genome-wide significant signals by GWAS is challenging due to the lack of statistical power. This is often addressed by increasing the sample size, either through genotyping more samples or using meta-analysis approaches^38^. Our enrichment pipeline integrates methylation information with GWAS-SS to identify novel risk SNPs that influence methylation at CpGs that would otherwise require larger GWASs to identify. The BrCa GWAS-SS^18^ used in this study reported 167 variants associated with BrCa risk. Of the 77 BrCa CpGs identified through the stepwise enrichment pipeline, 36 CpGs were present within 1 Mb of 30 out of the 167 risk variants. The latest BrCa GWAS^39^ comprises 133,384 cases and 113,789 controls, an increase of 10 and 9% in cases and controls, respectively, compared to the GWAS meta-analysis used in our study. The authors reported 22 additional risk variants, thus, summing to 189 variants associated with BrCa risk. In line with our expectations, we found an additional seven (total 43) of the 77 CpGs were present within 1 Mb of 35 out of the 189 risk variants (Supplementary Data 28). We also found a similar increase in CpGs near the latest PrCa GWAS variants. The PrCa GWAS-SS^24^ used in the enrichment pipeline reported 146 PrCa risk variants for men of European ancestry. Of the 81 PrCa CpGs identified, 47 were present within 1 Mb of 35 out of the 146 risk variants. The latest trans-ancestry GWAS meta-analysis for PrCa^40^ comprises approximately two-fold more cases and controls (107,247 cases and 127,006 controls including 85,554 cases and 91,972 controls of European ancestry) and reported 32 additional PrCa risk variants for men of European ancestry, thus, summing to 178 PrCa risk variants for European men. We found an additional two (total 49) of the 81 PrCa CpGs were present within 1 Mb of 43 out of 178 risk variants (Supplementary Data 29). Furthermore, the identified cancer-associated CpGs can guide the search for additional risk variants. Through approximate conditional analysis of the meQTL SNPs of the cancer-associated CpGs, we discovered several putative multi-SNP associations in both cancers (Supplementary Data 21 and 22). Of note are the SNPs on 3p21.31, 11q13.4 and 17q25.3 for BrCa and SNPs on 17p11.2 (Tables 3 and 4) for PrCa. The SNPs in these regions showed increased association significance compared to single-SNP analyses, suggesting that these SNPs may be identified as genome-wide significant signals in larger GWASs and may be important modifiers of risk at these genomic loci. Taken together, these results provide compelling evidence for the utility of our approach to discover robust novel risk loci and biomarkers by leveraging information from multiple omic datasets without further genotyping and sequencing.

Second, although GWAS have successfully identified hundreds of risk loci for BrCa and PrCa, identifying the mechanistic link between the variants and disease remains a challenge. Our results provide evidence for molecular links between the genetic sequence variants, DNA methylation, gene expression, and cancer risk. For example, the hypomethylation of the BrCa DM CpG cg15699386 (on 1q22 in the intronic region of *MUC1*) increases the risk of BrCa and (as seen in the i-MeWAS and conventional differential methylation analysis results) is consistent with the effect of its most significant predictor meQTL SNP (rs4971059) on BrCa risk. SNP rs4971059 is a robust BrCa risk SNP and the risk allele has a negative effect on methylation of the cg15699386 CpG. This is further supported by the negative correlation between the CpG and *MUC1* in our differential expression analysis and literature evidence, where overexpression of the gene is associated with increased BrCa risk^41^. Thus, it can be hypothesised that a functional consequence of rs4971059 is mediated by decreased methylation of the cg15699386 CpG which increases *MUC1* gene expression and thereby increases BrCa risk. A positive correlation between SNPs, CpG methylation and cancer risk was observed in 20 hypomethylated and nine hypermethylated CpGs in BrCa (Supplementary Data 30) and in 15 hypomethylated and 12 hypermethylated in PrCa (Supplementary Data 31). In addition, identification of moderate to strong LD (*r*^*2*^ > 0.4) between the genome-wide significant SNPs and the meQTL SNPs of 28 and 19 BrCa and PrCa CpGs, respectively, suggests that the genome-wide significant SNPs likely modulate cancer risk by influencing DNA methylation.

Our results have excellent potential to aid the clinical translation of BrCa and PrCa GWAS findings. For example, investigation of the DEGs associated with BrCa and PrCa CpGs using Drug Gene Interaction Database v4.2.0^42^, identified 74 and 31 genes that are potentially druggable genes in BrCa and PrCa, respectively (Supplementary Data 32 and 33). Common germline variants associated with overall BrCa survival is unknown^43^. In our approach, the i-MeWAS and TH-DM analyses identify the DM CpGs between the two extreme states (i.e., healthy and cancer), while TN-DM analysis identifies DM CpGs between cancer and NAT—which is often considered as an intermediate state between healthy and cancer^44^. Thus, suggesting that the enriched CpGs and genetic biomarkers are likely to be associated with both risk and progression. We interrogated the prognostic ability of the BrCa CpGs using their methylation status and overall survival data from the TCGA BrCa dataset with the MethSurv tool^45^ and identified that the methylation values of 36 BrCa CpGs were significant predictors of overall survival after correcting for age, clinical stage of the tumour, and oestrogen receptor status (FDR < 0.05; Supplementary Data 34). Successful replication of these results in independent datasets could enable clinical applications such as blood-based tests for diagnosis and prognosis.

The cross-cancer overlap analyses of the genome-wide significant DM CpGs from imputation-based and conventional differential methylation analyses implicated hypermethylation at the cg08129017 CpG in the Sterol Regulatory Element Binding Transcription Factor 1 (*SREBF1*) gene (17p11.2), which has not been previously associated with either cancer via GWAS analysis. *SREBF1* encodes a transcription factor that binds to the sterol regulatory element-1 (SRE1) and is involved in sterol synthesis and lipid metabolism. Emerging evidence shows that dysregulated lipid metabolism is a metabolic hallmark of cancer and increased lipid uptake is required for tumourigenesis, progression and metastasis^46^. Thus, increased expression of *SREBF1* can aid in the increased demands of lipids for tumour cell proliferation. Furthermore, upregulation of *SREBF1* is reported to be correlated with poor prognosis in BrCa and PrCa^47,48^ and hence, a potential therapeutic target to treat both cancers^49^. Over expression of *SREBF1* has also been implicated in other cancers (e.g., ovarian, pancreatic and glioma) and has been found to facilitate invasion^50–52^. Although hypermethylation at cg08129017 was consistently associated with BrCa and PrCa in our analyses, our differential expression analyses found *SREBF1* to be significantly upregulated in both BrCa and PrCa—which contradicts the classical view where DNA methylation is associated with suppression of gene expression. However, there is a growing body of evidence for a more complex relationship between DNA methylation and gene expression, including a significant proportion of hypermethylated genes associated with upregulated gene expression^53,54^. These studies, combined with our results, provide strong support for further BrCa and PrCa research and clinical applications involving *SREBF1*, for example, using targeted DNA demethylating therapy^55^. The eight cross-cancer CpGs were annotated to genes involved in transport (*KCNA3*, *KCNAB3* and *TRAPPC1*), signal transduction (*AGAP3* and *LIME1*), microtubule assembly (*FES* and *SHROOM1*), and metal binding (*FURIN* and *AGAP3*). Aberrant DNA methylation of ion channel genes (*KCNA3* and *KCNAB3*) and subsequent dysregulation of their expressions are known to contribute to carcinogenesis^56^, and suggested as potential targets for therapy^57^. The *FES* gene, a proto-oncogene, is frequently amplified in BrCa^58,59^ and its overexpression is attributed to tumour growth, angiogenesis and metastasis^60^. It is also an indicator of recurrence in PrCa^61^. Multiple lines of evidence exist for the oncogenic activities of *FURIN* and its potential for targeted therapy^62–64^.

A recent study conducted by Yang et al. also analysed the association between genetically influenced DNA methylation at CpGs and BrCa risk^65^. We identified eight BrCa CpGs overlapping with their CpG set. Another study by Wu et al. analysed the association between genetically influenced DNA methylation at CpGs and PrCa risk^66^. Here, we identified 15 PrCa CpGs overlapping with the CpGs identified in their study. The small overlap between the results is likely due to the following two reasons. First, the discrepancy in the genetically influenced CpGs analysed—we analysed 72,531 and 71,933 CpGs for BrCa and PrCa, respectively, while Yang et al. analysed 62,938 CpGs for BrCa and Wu et al. analysed 77,243 CpGs for PrCa. The second relates to important differences in the analysis pipelines. In our study, after the identification of DM CpGs via imputation-based i-MeWAS, we find additional evidence for DM of the CpGs in tumour tissues, whereas the Wu et al. and Yang et al. studies identified CpGs based solely on blood-tissue-based imputation.

Our study has two primary limitations. First, the methylation prediction models used in i-MeWAS are based on blood and not cancer-associated tissue. Indeed, the use of prediction models developed using methylation data from disease-relevant tissues will likely detect tissue-specific and further cancer-associated CpGs. However, the current TCGA dataset(s) lack sufficient power to develop accurate genetic prediction models. For example, there are <150 prostate tumour samples of European ancestry with genotype and methylation data in TCGA, whereas the blood-based prediction models used in this study were developed using genotype and methylation data from 533 healthy individuals of European ancestry. There is evidence for the presence of consistent meQTLs across various tissues^67^ and earlier i-MeWAS studies have shown that blood could be used as a proxy tissue to capture the CpG signature^68,69^. In addition, the integration of observed methylation data from tumour tissues in our approach aids to mitigate the spurious associations due to the heterogeneous nature of blood while identifying a robust and reliable set of implicated CpGs. Another limitation concerns the availability of methylation data from prostate tumour and NAT tissue samples. A larger sample size of the observed methylation dataset could have enabled improved enrichment estimation and further exploration of the functional role of hypomethylation in PrCa (e.g., via pathway analysis).

In conclusion, we demonstrated the application of our pipeline in breast and prostate cancers and identified novel putative loci, biomarkers and genes associated with cancer risk. The results provide evidence for common genetic and methylation influences shared between the two cancers. Our pipeline can be applied for diverse cancers and cancer-related traits such as survival. Such analyses can improve our understanding of the role of the common variants and DNA methylation in oncogenesis and progression, and offer opportunities for further targeted research and clinical application, particularly via blood-based tests for cancer.

## Methods

### Studies and quality control

#### GWAS studies

The GWAS summary statistics (GWAS-SS) for BrCa (122,977 cases and 105,974 controls of European ancestry) and PrCa (79,148 cases and 61,106 controls of European ancestry) were used in this study. For more details on the sample characteristics and meta-analysis, refer to refs. ^18,24^. Information including SNP id (in the form of b37 chromosome:basepair location), effect allele, and effect size (β) was extracted for analysis with EstiMeth and FUSION.

#### DNA methylation datasets

DNA methylation data measured using Illumina Infinium Human Methylation 450 K BeadChip in TCGA and GEO Omnibus studies for BrCa and PrCa were downloaded from the EWAS Data Hub database^21^ (https://bigd.big.ac.cn/ewas/datahub; download date: May 20, 2020). The data was downloaded from the EWAS Data Hub database as it hosts curated data. The curation steps performed by EWAS Data Hub to normalise and remove batch effects included - (i) normalisation of Type I probes among the arrays, (ii) bias correction associated with the technical difference between Type I and Type II array designs using Beta-Mixture Quantile Normalization (BMIQ) method^70^, (iii) removal of CpGs with high detection *P* (*P* > 2.2 × 10^−16^) and (iv) removal of samples with more than 20% of the CpGs with high detection *P* values. For more details on the data curation performed by EWAS Data Hub, refer to ref. ^21^.

The TCGA^19^ study for BrCa included methylation measurements of breast tumour and histologically normal tissue adjacent to the tumour (NAT) samples. The GSE101961^20^ study included methylation measurements of healthy breast samples. The samples from both datasets were restricted to Caucasian females to ensure population similarity with the GWAS data. For PrCa analysis, prostate tumour and NAT samples from TCGA^13^ and GSE76938^25^ studies were downloaded. Here, the samples were restricted to Caucasian males to match the GWAS population. After downloading the samples, we performed further quality control for both BrCa and PrCa. This included the removal of CpGs with missing values in more than 10% of the samples in the tumour, NAT and healthy samples^71^. Missing values for the remaining selected CpGs were given the median values^72^. Lastly, we conducted PCA analysis to identify outlier samples. We detected outlier samples in the PrCa dataset (Supplementary Fig. 1). The box and whiskers plot of principal component 1 values was used to systematically remove the outlier samples in each category (TCGA tumour, TCGA NAT, GSE76938 tumour and GSE76938 NAT samples) (Supplementary Fig. 2). The final BrCa methylation dataset analysed in this study included 499, 91, and 81 tumour, NAT, and healthy samples, respectively, while the PrCa dataset included 189 and 82 tumour and NAT samples, respectively.

#### Gene expression datasets

RNASeq gene expression datasets from the TCGA study for BrCa and PrCa were downloaded from the Toil RNASeq recompute compendium^73^, hosted by the UCSC Xena browser^74^. Only those samples matching the tumour and NAT samples in the TCGA DNA methylation dataset were retained for differential gene expression analysis which resulted in 497 breast tumour and 78 NAT samples for BrCa, and 134 prostate tumour and 26 NAT samples for PrCa.

### Differential methylation using i-MeWAS

To identify the DM CpGs through the i-MeWAS approach, we used the EstiMeth R package^17^. The package can impute differential methylation of 86,518 genetically influenced CpGs using meQTL SNP-based prediction models. The prediction models were built using DNA methylation and genotype data profiled from blood samples of 533 healthy young adults of European ancestry. A prediction model for each CpG is built using linear regression with elastic net regularisation fitted between the DNA methylation intensity and the common *cis* SNPs associated with the CpG. Common *cis* SNPs are SNPs with minor allele frequency >0.5 and present within 1 Mb from the CpG. In addition, only those SNPs that were overlapping with the 1000 Genome Phase 3 reference panel for the European population (*N* = 503) were used in the model building.

To impute the differential methylation Z score (i.e., the differential methylation association score), the function MetaMethScan available in the package was used. The function implements an imputation approach similar to the MetaXcan approach^75^ which is the linear combination of the SNP weights, SNP covariance structure (linkage disequilibrium (LD) matrix), and the GWAS effect size to estimate the differential methylation Z score. The 1000 Genome Phase 3 reference panel for the European population was used to estimate LD between SNPs. The GWAS-SS of BrCa and PrCa were provided as input to predict the genetically influenced DM CpGs. Following the estimation of the differential methylation Z scores of the CpGs, the *P* values for the Z scores based on normal distribution were adjusted for multiple hypothesis testing using the Benjamini-Hochberg method implemented in the “p.adjust” function in R. CpGs with FDR < 0.05 were considered statistically significant DM CpGs associated with cancer risk.

### Differential methylation using experimental data

We performed two differential methylation analyses using the experimental DNA methylation data (i) tumour vs healthy samples (TH-DM) and (ii) tumour vs NAT samples (TN-DM). For both analyses, DM CpGs were detected using linear regression modelling as follows:

1 $${DNA}\,{methylation}\,\left(\beta \,{value}\,{of}\,a\,{CpG}\,{site}\right) \sim {Sample}\,{type}+{Age}+{{Age}}^{2}\\ +{Top}\,{principal}\,{components}$$

where the tumour and healthy samples were coded as case and control, respectively, for *sample type* in the TH-DM analysis, while tumour and NAT samples were coded as case and control, respectively, for *sample type* in the TN-DM analysis. We used the first ten and five principal components in the model for BrCa and PrCa, respectively. The significance values (*P*) obtained for the coefficient estimate for *sample type* were adjusted using the Benjamini–Hochberg method and CpGs with FDR < 0.05 were considered statistically significant DM CpGs.

### Stepwise enrichment analysis

Stepwise overlaps between i-MeWAS, TH-DM and TN-DM results were estimated, and the overlap significance was assessed using a one-sided binomial test. In the first iteration (Fig. 1, Iteration 1), the overlap between i-MeWAS and TH-DM is tested. Here, the null hypothesis is that the proportion of overlapping DM CpGs from i-MeWAS is equal to the observed proportion of DM CpGs in TH-DM analysis. We reject the null hypothesis when the proportion of overlapping CpGs is greater than the observed proportion at *P* < 0.05 and proceed to conduct the second iteration (Fig. 1, Iteration 2). Here, the null hypothesis is that the proportion of overlapping CpGs from Iteration 1 with TN-DM analysis is equal to the observed proportion of DM CpGs in TN-DM analysis. When the evidence for enrichment is statistically significant (*P* < 0.05), we selected the set of overlapping CpGs from Iteration 2 as the cancer-associated CpGs. In PrCa analysis, sufficient samples for healthy prostate were unavailable; hence, enriched CpGs were identified through overlap analysis between i-MeWAS and TN-DM results.

### Location and functional annotation of CpGs

The ANNOVAR software tool^29^ was used to annotate the selected CpGs to genes and their genomic location such as exonic, intronic, 5’-UTR, 3’-UTR, intergenic, splicing (when a variant is within 2 bp of a splicing junction), upstream, and downstream (CpG overlaps 1 kb region upstream and downstream, respectively, of transcription start site). The eForge v2.0 tool^23^ along with the Consolidated Roadmap Epigenomics data^76^ was used to assess the enrichment of the CpGs in histone modification marks (H3K4me3, H3K4me1, H3K27me3, H3K36me3 and H3K9me3).

### Differential gene expression analysis

Candidate genes for differential expression analysis were chosen using three approaches: (i) ANNOVAR, (ii) distance-based and (iii) eQTL-based. The ANNOVAR approach used ANNOVAR software^29^ to annotate genes to the enriched CpGs. The annotated genes were tested for differential expression using the DESeq2 R package with default parameter setting^26^ which included the removal of genes with less than ten counts. After correcting the *P* values for multiple testing using the Benjamini–Hochberg approach, genes with FDR < 0.05 were selected as DEGs.

In the distance-based approach, we examined the DEGs present within varying flanking distances around a CpG to identify the candidate gene set. Cut-offs from 1 kb to 10 Mb were used to define the genomic windows. Differential expressions of the genes present (including partially present genes) within each window were tested using DESeq2 with default parameter setting and the *P* values were corrected for multiple testing using the Benjamini-Hochberg method. Genes with FDR < 0.05 were selected as DEGs in each window. Next, enrichment of DEGs in each genomic window was estimated using the one-sided exact binomial test with genome-wide differential expression rate as the null proportion (Null_BrCa_ = 0.492, Null_PrCa_ = 0.302). The gene set with maximum decrease in enrichment when increasing the cut-off distance was chosen as the most likely DEGs associated with the enriched CpGs.

Lastly, an eQTL-based approach was used to determine genetically regulated genes associated with the enriched CpGs. Using the methylation prediction models (obtained from EstiMeth) and the gene expression prediction models (obtained from FUSION^27^), we associated a gene to a CpG if the SNPs that were used for the gene expression prediction (eQTL SNPs) were significantly enriched with meQTL SNPs of that CpG. As expression prediction models are available at the tissue level, we examined independently whole blood and breast tissue for BrCa, and whole blood and prostate tissue for PrCa. Enrichment was tested using a one-sided binomial test and the null proportion was defined as the median SNP overlap proportion in genes that have at least one overlapping SNP (Null_BrCa, Breast_ = 0.009202, Null_BrCa,Whole blood_ = 0.008972, Null_PrCa, Prostate_ = 0.009852, Null_PrCa, Whole blood_ = 0.010399). Following correction for multiple testing using the Benjamini–Hochberg method, genes with binomial test significance FDR < 0.05 were selected. Differential expression of the selected genes was tested using the FUSION software^27^. The input to FUSION includes the genetic prediction models of the selected genes and cancer GWAS summary results. Multiple testing correction was done using the Benjamini–Hochberg method and genes with FDR < 0.05 were selected as the differentially expressed genes.

### Mediation analysis

To determine if the enriched CpGs were involved in transcription regulation, we tested the conditional differential expression of the CpG-associated genes in tumour vs NAT samples given the methylation status of the CpG. For this analysis, we used the log_2_ rsem normalised gene expression data for tumour and NAT samples for BrCa and PrCa provided by TCGA. The gene expression data was downloaded from the UCSC Xena browser. The conditional differential expression of a gene was tested using linear regression as follows:

2 $${Gene}\,{Expression} \sim {SampleTy}{pe}+{CpG}\,{methylation}\\ \,\,\,\,+{SampleType}* {CpG}\,{methylation}$$

where *Gene Expression* refers to log_2_ rsem normalised expression of the gene, *SampleType* refers to whether the sample is tumour or NAT, *CpG methylation* refers to the methylation level (beta value) of the associated CpG, and the interaction term SampleType*CpG methylation refers to the conditional association of the gene expression to sample type given the CpG methylation. Genes with statistically significant SampleType*CpG methylation term after correction for multiple testing (conditional association FDR < 0.05) were considered significantly differentially expressed genes given CpG methylation.

### Pathway analysis of genes

The functional implications of the different gene sets identified in this study were investigated through pathway analysis with Enrichr (https://amp.pharm.mssm.edu/Enrichr/)^28,33^. It performs statistical enrichment using Fisher’s exact test and the *P* values obtained from the tests were adjusted for multiple testing using the Bonferroni method. The pathway databases that were examined include the NCATS BioPlanet 2019, Elsevier, Kyoto Encyclopedia of Genes and Genomes (KEGG) 2019, Human WikiPathways 2019, MSigDB and Reactome. In all the databases, only those pathways with at least two overlapping genes were selected.

### Contribution of meQTL SNPs to the familial relative risk of breast and prostate cancers

We estimated the proportion of breast and prostate cancer familial risk contributed by the meQTL SNPs associated with BrCa and PrCa CpGs using a log-additive model provided in ref. ^18^:

3 $$\mathop{\sum}\limits_{i} {p}_{i}\left(1-{p}_{i}\right) ( {\beta }_{i}^{2}-{\tau }_{i}^{2})/{{{{{\rm{ln}}}}}}(\lambda )$$

where _*pi*_ is the minor allele frequency for the meQTL SNP *i*, *β*_*i*_ is the log(odds ratio) or effect size for the meQTL SNP *i* in the relevant cancer GWAS, *τ*_*i*_ is the standard error of *β*_*i*_, and *λ* represents the overall familial relative risk. For BrCa *λ* = 2 and for PrCa *λ* = 2.5.

### Identification of independent SNPs using conditional analysis

Among the enriched CpGs, we selected those CpGs with no genome-wide significant SNPs (*P*_*GWAS*_ < 5 × 10^−8^, *P*_*GWAS*_ obtained from the GWAS summary results) in ±1 Mb flanking genomic regions. For the selected CpGs, we conducted an iterative conditional analysis of the predictor SNPs (meQTLs SNPs) using the genome-wide complex trait analysis (GCTA) software (version 1.93.2)^35^. To avoid false-positive results, for two or more of the selected CpGs present within 1 Mb of each other, we analysed the meQTL SNPs of these CpGs together. We begin by choosing the meQTL SNP with the smallest *P*_*GWAS*_ value less than the threshold = 0.05. The association significances of the remaining meQTL SNPs are calculated conditioning on the selected meQTL SNP. Next, a new meQTL SNP with the lowest conditional *P* (*P*_*cond*_) value less than the threshold value is selected and we perform a second iteration of conditional analysis along with the newly selected meQTL SNP. We repeat the steps until no meQTL SNP can be selected. To avoid multicollinearity, the meQTL SNPs in high LD (*r*^2^ >0.9) with previously selected meQTL SNPs are not chosen. The 1000 Genome Phase 3 reference panel for the European population, downloaded from https://ctg.cncr.nl/software/magma^77^, was provided to estimate LD. The final list of selected independent meQTLs was chosen as the novel secondary loci contributing to cancer via methylation regulation.

### GWAS Meta-analysis

We meta-analysed the BrCa and PrCa GWAS-SS using the GWAMA software^37^ using an inverse-weighted fixed-effect model. The combined associations for 11,784,678 unique imputed and genotyped SNPs present in both GWASs were estimated. The Cochran’s Q heterogeneity statistic and the associated *P* values (*P*_het_) for each SNP were also estimated using the GWAMA software during the meta-analysis.

### Statistics and reproducibility

All statistical analyses including binomial tests and multiple testing corrections were conducted in R version 3.6.3.

### Reporting summary

Further information on research design is available in the Nature Research Reporting Summary linked to this article.

## Supplementary information

Supplementary Information

Description of Additional Supplementary Files

Supplementary Data 1-20

Supplementary Data 21

Supplementary Data 22

Supplementary Data 23-34

Reporting summary

## Data Availability

All datasets used in the analysis are publicly available. The BrCa GWAS summary results are available at http://bcac.ccge.medschl.cam.ac.uk/ and the PrCa GWAS summary results are available at http://practical.icr.ac.uk/blog/?page_id=8164. The curated DNA methylation datasets for both cancers (TCGA, GSE101961, and GSE76938) are available at the EWAS Data Hub database (https://bigd.big.ac.cn/ewas/datahub)^21^. The TCGA gene expression datasets for both cancers are available at the UCSC Toil RNAseq recompute compendium^73^ (https://xenabrowser.net/datapages/?hub=https://toil.xenahubs.net:443). The analysis scripts can be provided by the corresponding authors upon request.
