# Supplementary material for: Integrative multi-omic analysis identifies genetically influenced DNA methylation biomarkers for breast and prostate cancers
Source: Commun Biol. 2022 Jun 16;5:594. doi: 10.1038/s42003-022-03540-4 (PMC9203749; doi:10.1038/s42003-022-03540-4)
Supplement: Supplementary file 1 — Supplementary Information [file 42003_2022_3540_MOESM1_ESM.pdf]

Integrative multi-omic analysis identifies genetically influenced DNA methylation biomarkers for breast and prostate cancers

Supplementary Figure 1: PCA analysis of breast cancer and prostate cancer methylation datasets

a) PCA of breast cancer dataset

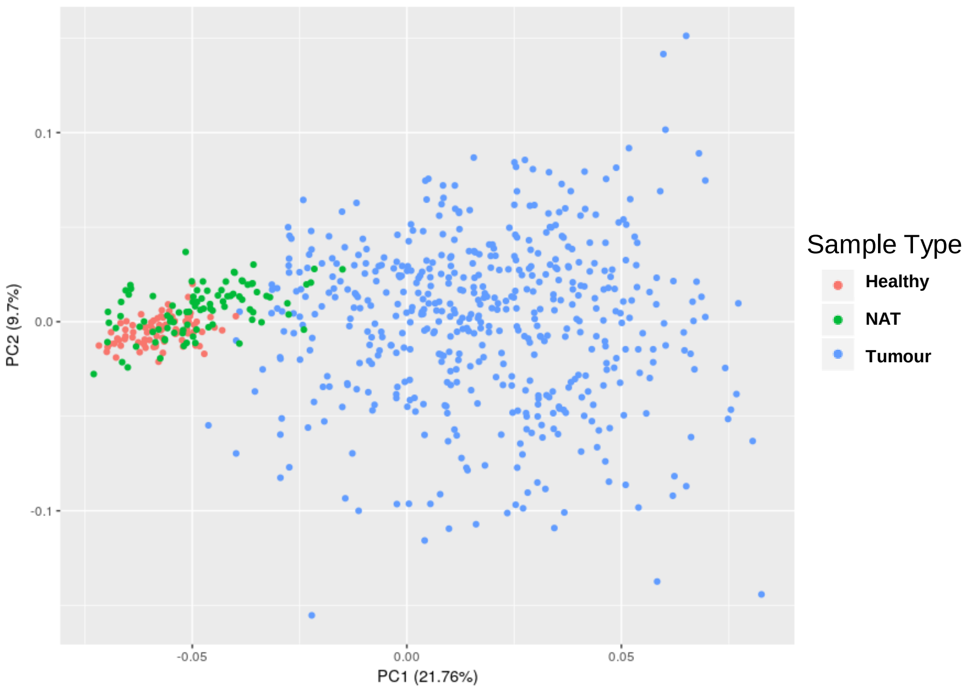

b) PCA of prostate cancer dataset

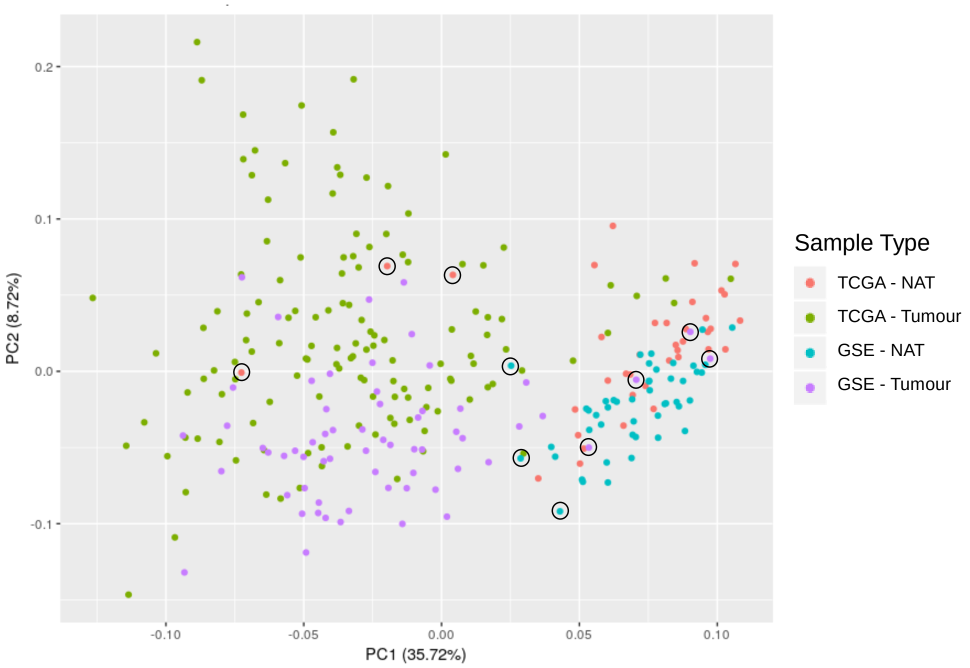

Supplementary Figure 2: Box whisker plot of principal component 1 of prostate cancer samples.

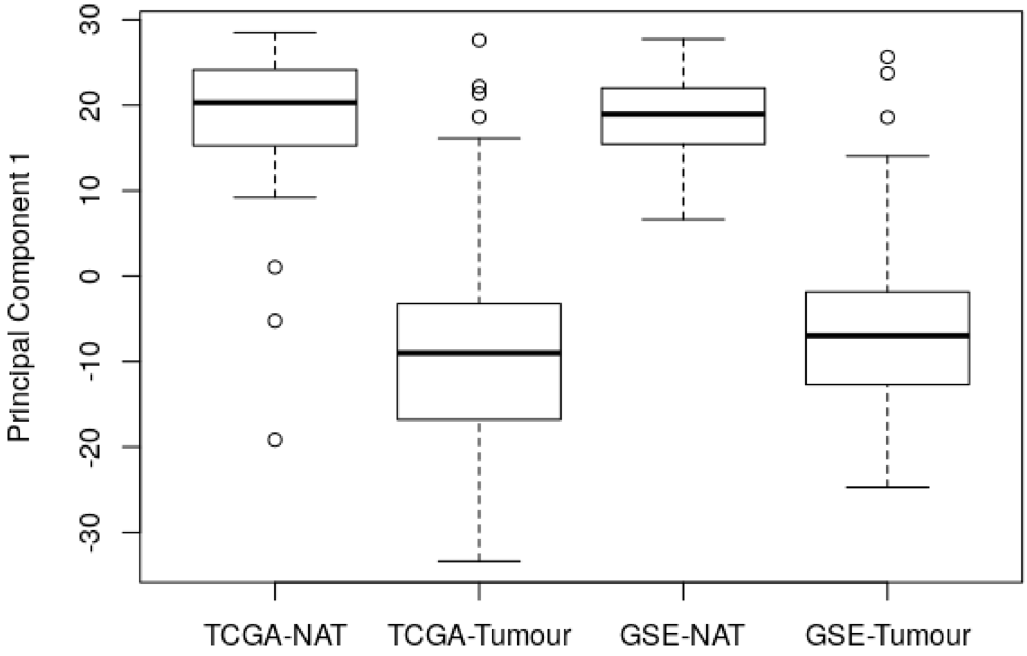

Notes: Dots represent the outlier samples which were removed

Supplementary Figure 3: Enriched histone marks in breast cancer CpGs across various tissues and cell types

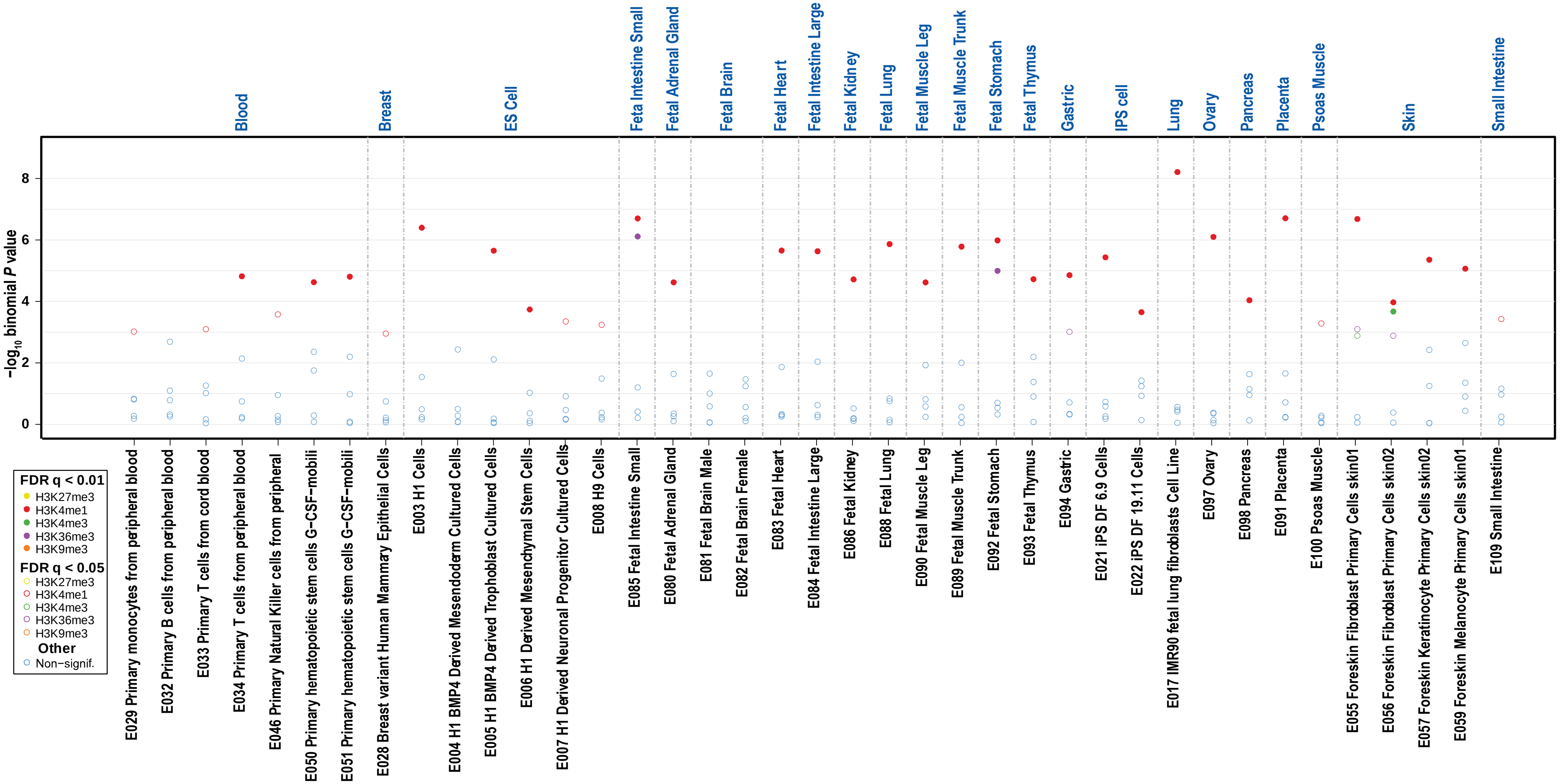

Supplementary Figure 4: Enriched H3K4me1 marks in identified prostate cancer CpGs across various tissues and cell types

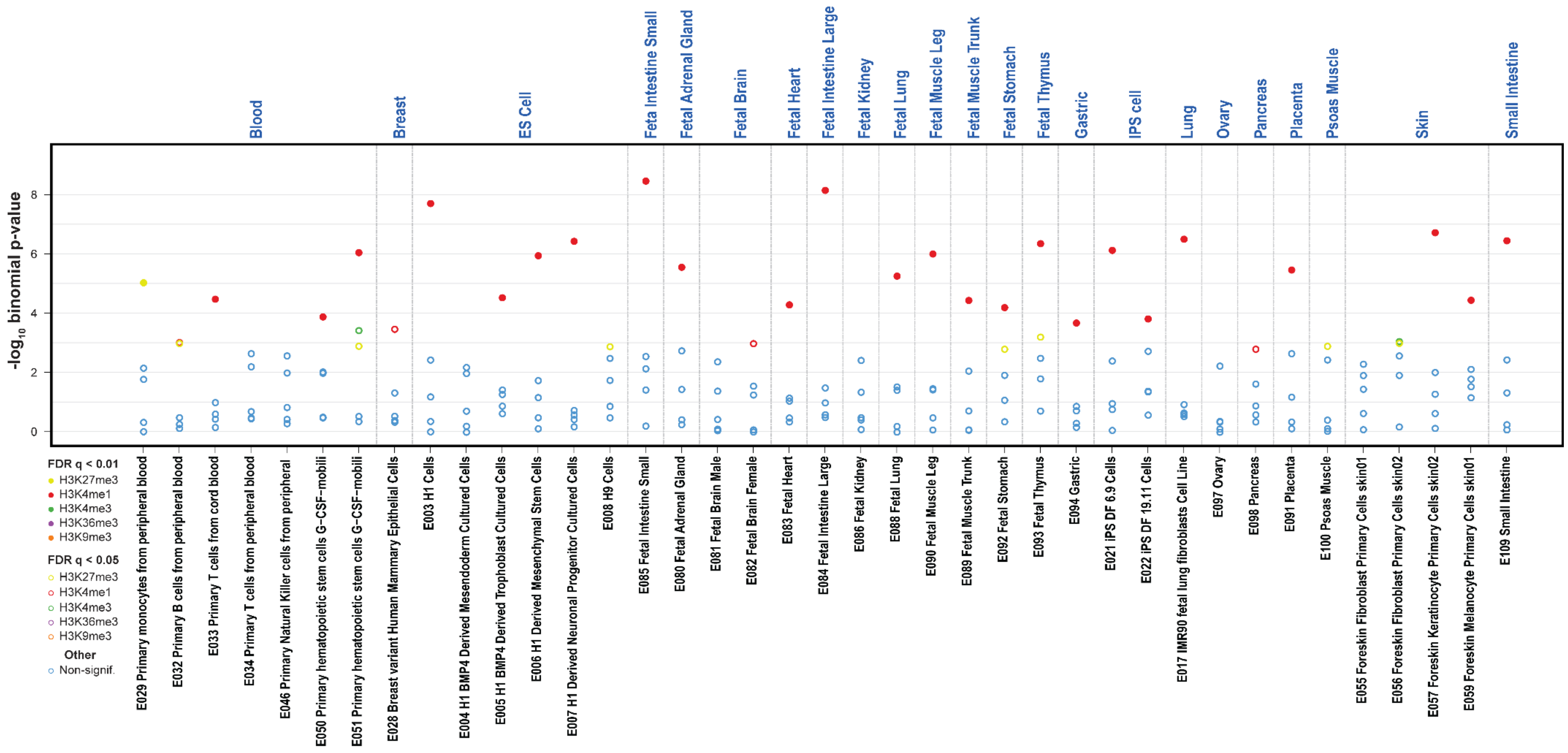

Supplementary Figure 5: Locuszoom plots of ±1Mb flanking regions of pleiotropic genetically influenced DM CpGs. The association significances (*P* values) of SNPs were obtained from meta-analysed BrCa+PrCa GWAS-SS

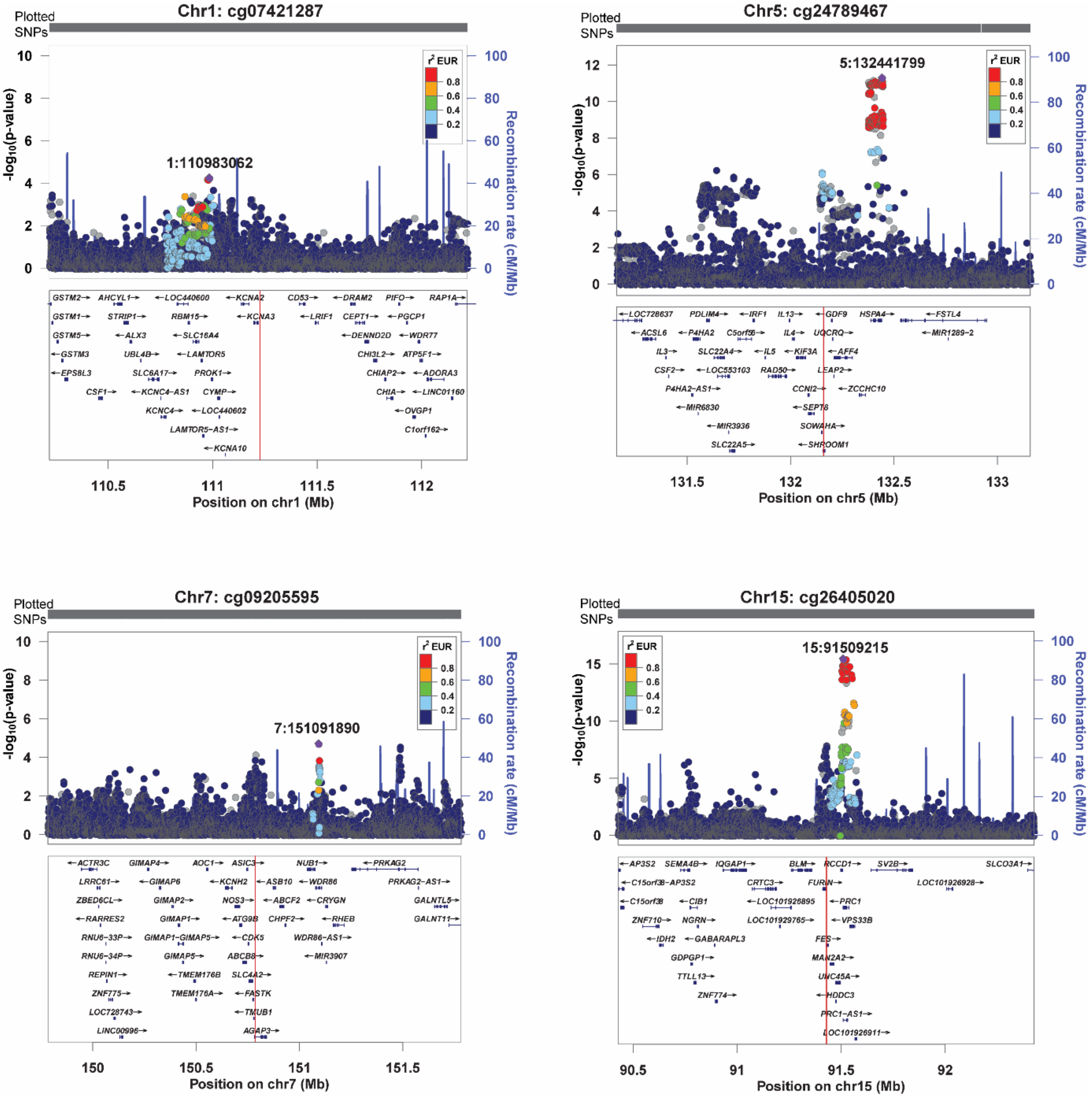

(Cont.)

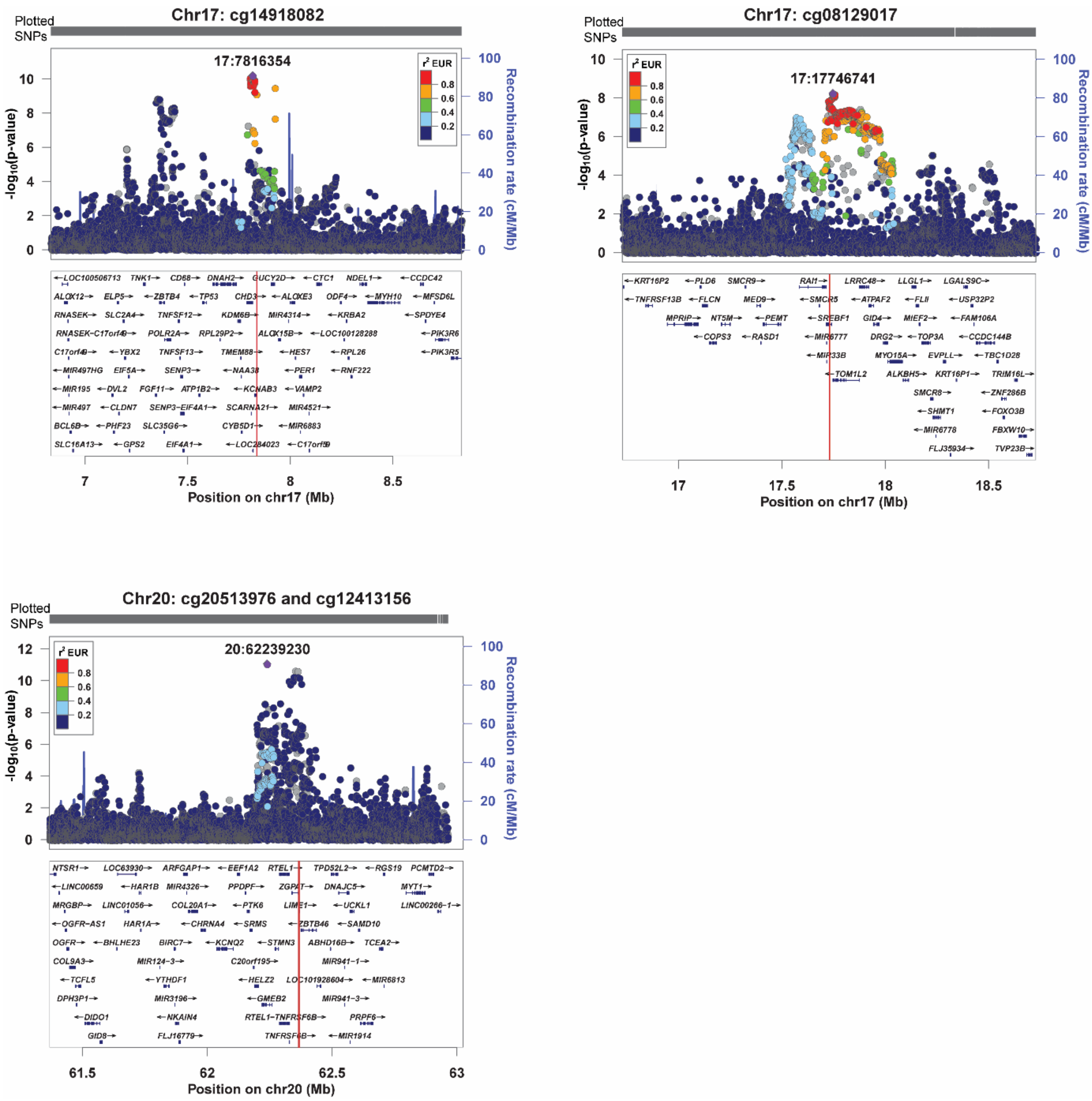
Chr17: cg14918082
Plotted SNPs
17:7816354
-log10(p-value)
Recombination rate (cM/Mb)
r² EUR
Position on chr17 (Mb)
Chr17: cg08129017
Plotted SNPs
17:17746741
-log10(p-value)
Recombination rate (cM/Mb)
r² EUR
Position on chr17 (Mb)
Chr20: cg20513976 and cg12413156
Plotted SNPs
20:62239230
-log10(p-value)
Recombination rate (cM/Mb)
r² EUR
Position on chr20 (Mb)
