# Supplementary material for: Integrative multi-omic analysis identifies genetically influenced DNA methylation biomarkers for breast and prostate cancers
Source: Commun Biol. 2022 Jun 16;5:594. doi: 10.1038/s42003-022-03540-4 (PMC9203749; doi:10.1038/s42003-022-03540-4)
Supplement: Supplementary file 2 — Description of Additional Supplementary Files [file 42003_2022_3540_MOESM2_ESM.pdf]

Description of Additional Supplementary Files

File name: Supplementary Data 1
Description: BrCa CpGs identified using the bioinformatics pipeline

File name: Supplementary Data 2
Description: Distribution of identified BrCa CpGs across genomic regions, CpG islands, and chromosomes

File name: Supplementary Data 3
Description: Prca CpGs identified using the bioinformatics pipeline

File name: Supplementary Data 4
Description: Distribution of identified PrCa CpGs across genomic regions, CpG islands, and chromosomes

File name: Supplementary Data 5
Description: Differentially expressed genes based on ANNOVAR annotation associated with BrCa CpGs

File name: Supplementary Data 6
Description: Mediation analysis results for ANNOVAR-based genes associated with BrCa CpGs

File name: Supplementary Data 7
Description: Differentially expressed genes based on ANNOVAR annotation associated with PrCa CpGs

File name: Supplementary Data 8
Description: Mediation analysis results for ANNOVAR-based genes associated with PrCa CpGs

File name: Supplementary Data 9
Description: Enrichment results of distance-based differentially expressed genes associated with BrCa CpGs

File name: Supplementary Data 10
Description: Differentially expressed genes located within 25kb of BrCa CpGs

File name: Supplementary Data 11
Description: Mediation analysis results for distance-based genes (genes with ±25kb flanking window) associated with BrCa CpGs

File name: Supplementary Data 12
Description: Enrichment results of distance-based differentially expressed genes associated with PrCa CpGs

File name: Supplementary Data 13
Description: Differentially expressed genes located within 10kb of PrCa CpGs

File name: Supplementary Data 14
Description: meQTL SNPs enriched-genetically influenced genes that were differentially expressed in breast tissue and whole blood

File name: Supplementary Data 15
Description: meQTL SNPs enriched-genetically influenced genes that were differentially expressed in prostate tissue and whole blood

File name: Supplementary Data 16
Description: Genomewide enrichment results of hyper- and hypo-methylated CpGs in BrCa and PrCa

File name: Supplementary Data 17
Description: Site-specific enrichment analysis of hypermethylated CpGs

File name: Supplementary Data 18
Description: Pathways enriched by DEGs associated with enriched hypomethylated CpGs in breast cancer and prostate cancer

File name: Supplementary Data 19
Description: GWAS SNPs around BrCa CpGs

File name: Supplementary Data 20
Description: GWAS SNPs around PrCa CpGs

File name: Supplementary Data 21
Description: Iterative conditional analysis of the meQTL SNPs of BrCa CpGs identified in suggestive and novel regions

File name: Supplementary Data 22
Description: Iterative conditional analysis of the meQTL SNPs of PrCa CpGs identified in suggestive and novel regions

File name: Supplementary Data 23
Description: LD between selected meQTL SNPs of BrCa CpGs in genome-wide significant regions and genome-wide significant SNP

File name: Supplementary Data 24
Description: LD between selected meQTL SNPs of PrCa CpGs in genome-wide significant regions and genome-wide significant SNP

File name: Supplementary Data 25
Description: Pairwise overlap to identify common cross-cancer CpGs

File name: Supplementary Data 26
Description: Most significant SNPs around the eight overlapping CpGs in BrCa and PrCa GWAS

File name: Supplementary Data 27
Description: Association results for the predictor SNPs of cg08129017 in breast cancer, prostate cancer, and meta-analysis

File name: Supplementary Data 28
Description: BrCa CpGs found within 1Mb of known and novel BrCa risk loci

File name: Supplementary Data 29
Description: PrCa CpGs found within 1Mb of known and novel PrCa risk loci

File name: Supplementary Data 30
Description: Direction of influence of GWAS SNPs and the BrCa CpGs on BrCa

File name: Supplementary Data 31
Description: Direction of influence of GWAS SNPs and the PrCa CpGs on PrCa

File name: Supplementary Data 32

Description: BrCa CpGs associated druggable genes identified from DGIdb

File name: Supplementary Data 33
Description: PrCa CpGs associated druggable genes identified from DGIdb

File name: Supplementary Data 34
Description: Survival analysis of BrCa CpGs using MethSurv tool. Survival analysis was conducted for each of the enriched BrCa CpG using Cox regression models. Covariates in the model included age, clinical stage, and ER status
