# Supplementary material for: Integrative multi-omic analysis identifies genetically influenced DNA methylation biomarkers for breast and prostate cancers
Source: Commun Biol. 2022 Jun 16;5:594. doi: 10.1038/s42003-022-03540-4 (PMC9203749; doi:10.1038/s42003-022-03540-4)
Supplement: Supplementary file 7 — Reporting summary [file 42003_2022_3540_MOESM7_ESM.pdf]

nature portfolio

Corresponding author(s): Anita Sathyanarayanan and Dale R Nyholt

Last updated by author(s): Apr 29, 2022

Reporting Summary

Nature Portfolio wishes to improve the reproducibility of the work that we publish. This form provides structure for consistency and transparency in reporting. For further information on Nature Portfolio policies, see our Editorial Policies and the Editorial Policy Checklist.

Statistics

For all statistical analyses, confirm that the following items are present in the figure legend, table legend, main text, or Methods section.

n/a	Confirmed	
☐	☒	The exact sample size (*n*) for each experimental group/condition, given as a discrete number and unit of measurement
☐	☒	A statement on whether measurements were taken from distinct samples or whether the same sample was measured repeatedly
☐	☒	The statistical test(s) used AND whether they are one- or two-sided<br>*Only common tests should be described solely by name; describe more complex techniques in the Methods section.*
☐	☒	A description of all covariates tested
☐	☒	A description of any assumptions or corrections, such as tests of normality and adjustment for multiple comparisons
☐	☒	A full description of the statistical parameters including central tendency (e.g. means) or other basic estimates (e.g. regression coefficient) AND variation (e.g. standard deviation) or associated estimates of uncertainty (e.g. confidence intervals)
☐	☒	For null hypothesis testing, the test statistic (e.g. *F*, *t*, *r*) with confidence intervals, effect sizes, degrees of freedom and *P* value noted<br>*Give P values as exact values whenever suitable.*
☒	☐	For Bayesian analysis, information on the choice of priors and Markov chain Monte Carlo settings
☒	☐	For hierarchical and complex designs, identification of the appropriate level for tests and full reporting of outcomes
☐	☒	Estimates of effect sizes (e.g. Cohen's *d*, Pearson's *r*), indicating how they were calculated

Our web collection on statistics for biologists contains articles on many of the points above.

Software and code

Policy information about availability of computer code

Data collection	No software was used
Data analysis	R version 3.6.3, ANNOVAR, eFORGE v1.2, FUSION, GWAMA, GCTA software version 1.93.2

For manuscripts utilizing custom algorithms or software that are central to the research but not yet described in published literature, software must be made available to editors and reviewers. We strongly encourage code deposition in a community repository (e.g. GitHub). See the Nature Portfolio guidelines for submitting code & software for further information.

Data

Policy information about availability of data

All manuscripts must include a data availability statement. This statement should provide the following information, where applicable:

- Accession codes, unique identifiers, or web links for publicly available datasets
- A description of any restrictions on data availability
- For clinical datasets or third party data, please ensure that the statement adheres to our policy

All datasets used in the analysis are publicly available. The breast cancer GWAS summary results are available at http://bcac.ccge.medschl.cam.ac.uk/ and the prostate cancer GWAS summary results are available http://practical.icr.ac.uk/blog/?page_id=8164. The curated DNA methylation datasets for both cancers (TCGA, GSE101961, and GSE76938) are available at the EWAS Data Hub database (https://bigd.big.ac.cn/ewas/datahub)21. The TCGA gene expression datasets for both cancers are available at the UCSC Toil RNAseq recompute compendium67 (https://xenabrowser.net/datapages/?hub=https://toil.xenahubs.net:443). The analysis scripts can be provided by the corresponding authors upon request.

Field-specific reporting

Please select the one below that is the best fit for your research. If you are not sure, read the appropriate sections before making your selection.

☒ Life sciences ☐ Behavioural & social sciences ☐ Ecological, evolutionary & environmental sciences

For a reference copy of the document with all sections, see nature.com/documents/nr-reporting-summary-flat.pdf

Life sciences study design

All studies must disclose on these points even when the disclosure is negative.

Sample size	The GWAS summary statistics for breast cancer (BrCa) included 122,977 cases and 105,974 controls of European ancestry and prostate cancer (PrCa) included 79,148 cases and 61,106 controls of European ancestry.<br>TCGA BrCa methylation data: 499 Tumour and 91 normal tissue adjacent to the tumour (NAT) samples<br>GSE101961 healthy breast methylation: 81 samples<br>TCGA BrCa gene expression data: 497 tumour and 78 NAT samples<br>TCGA PrCa methylation data: 129 tumour and 35 NAT samples<br>GSE76938 PrCa methylation data: 60 tumour and 47 NAT samples<br>TCGA PrCa gene expression data: 134 tumour and 26 NAT samples
Data exclusions	Samples were restricted to european ancestry. For differential methylation analysis, outlier samples were removed based on PCA analysis.
Replication	The differentially methylated sites predicted using imputation based approach were validated using conventional differential methylation using TCGA observed datasets
Randomization	Not relevant to this study as this is an epidemiological study.
Blinding	Not relevant to this study as this is an epidemiological study.

Reporting for specific materials, systems and methods

We require information from authors about some types of materials, experimental systems and methods used in many studies. Here, indicate whether each material, system or method listed is relevant to your study. If you are not sure if a list item applies to your research, read the appropriate section before selecting a response.

Materials & experimental systems

n/a	Involved in the study	
☒	☐	Antibodies
☒	☐	Eukaryotic cell lines
☒	☐	Palaeontology and archaeology
☒	☐	Animals and other organisms
☐	☒	Human research participants
☒	☐	Clinical data
☒	☐	Dual use research of concern

Methods

n/a	Involved in the study	
☒	☐	ChIP-seq
☒	☐	Flow cytometry
☒	☐	MRI-based neuroimaging

Human research participants

Policy information about studies involving human research participants

Population characteristics	BrCa GWAS included 122,977 cases and 105,974 controls of European ancestry from studies collaborating in the Breast Cancer Association Consortium (BCAC), Discovery, Biology and Risk of Inherited Variants in Breast Cancer Consortium (DRIVE), Collaborative Oncological Gene-environment Study (iCOGS) and 11 other breast cancer genome-wide association studies.<br><br>PrCa GWAS included 79,148 cases and 61,106 controls of European ancestry included studies from the PRACTICAL consortium, BPC3, CAPS1, CAP2, UK Stage1, UK Stage 2, iCOGS and Pegasus.<br><br>The Cancer Genome Atlas program conducted multi-omic sequencing of several cancers including breast and prostate cancers. This study used methylation data of 590 breast cancer and 164 prostate cancer for differential methylation analyses. Also, gene expression data of 575 breast cancer and 260 prostate cancer.<br><br>GSE101961 measure DNA methylation in breast tissues from a cross-sectional study of 121 cancer-free women.<br><br>GSE76938 measured DNA methylation in 73 clinically-annotated fresh-frozen prostate tissues and 63 benign prostate tissues.

Recruitment	GSE101961 - women (n = 121) who underwent reduction mammoplasty at Georgetown University Medical Center (Washington, DC), the University of Maryland (College Park, MD), the Washington Hospital Center (Washington, DC) and the Center for Plastic Surgery (Buffalo, NY) provided written informed consent, an epidemiologic questionnaire, blood and their residual breast tissues.<br><br>GSE76938 - collected the prostate tissues used in this study at Stanford University Medical Center between 1999 and 2007 from patients undergoing radical prostatectomy
Ethics oversight	BrCa GWAS - All participating studies were approved by their appropriate ethics review board and all subjects provided informed consent. PMID: 29059683<br><br>PrCa GWAS - All studies were approved by the appropriate ethics committees (as described in the references for each study listed in Supplementary Table 1), and informed consent was obtained from all participants. PMID: 29892016<br><br>GSE101961 - women (n = 121) who underwent reduction mammoplasty provided written informed consent, an epidemiologic questionnaire, blood and their residual breast tissues. Institutional Review Boards was received at each institution. PMID: 29383109<br><br>GSE76938 - patient written informed consent under protocol number 13873 approved by the Stanford University IRB committee. PMID: 28412973

Note that full information on the approval of the study protocol must also be provided in the manuscript.
